# A convergent, umpoled synthesis of 2-(1-amidoalkyl)pyridines

**DOI:** 10.3762/bjoc.12.1

**Published:** 2016-01-04

**Authors:** Tarn C Johnson, Stephen P Marsden

**Affiliations:** 1 Institute of Process Research and Development, School of Chemistry, University of Leeds, Woodhouse Lane, Leeds LS2 9JT, UK

**Keywords:** azlactones, pyridines, pyridine *N*-oxides, substitution

## Abstract

A convenient, one-pot, two-component synthesis of 2-(1-amidoalkyl)pyridines is reported, based upon the substitution of suitably-activated pyridine *N*-oxides by azlactone nucleophiles, followed by decarboxylative azlactone ring-opening. The synthesis obviates the need for precious metal catalysts to achieve a formal enolate arylation reaction, and constitutes a formally ‘umpoled’ approach to this valuable class of bioactive structures.

## Introduction

**Figure 1:**
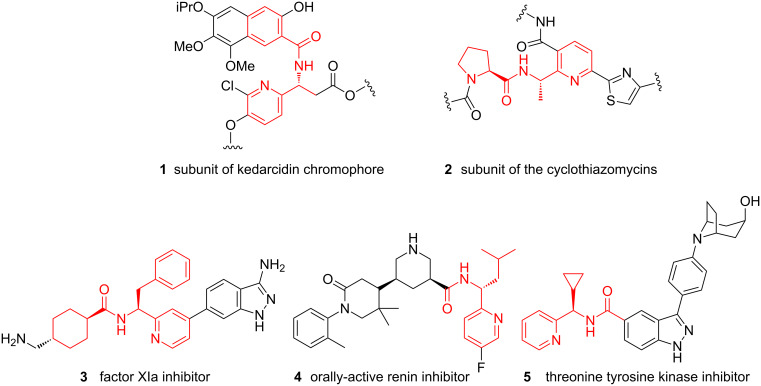
Examples of naturally-occurring and synthetic bioactive (amidoalkyl)pyridines.

Pyridines constitute the most frequently observed class of heterocycles found in pharmaceutical products [1]. As such, there is significant demand for synthetic methods that enable access both to structurally novel and diverse substituted pyridines for new medicines discovery, and for the development of clean, efficient and robust methods for their manufacture on large scale. Pyridines bearing a 1-amidoalkyl substituent at the 2-position are found in numerous biologically active natural products such as the antitumour antibiotic kedarcidin chromophore **1** [2] and the RNA polymerase inhibitor cyclothiazomycin B1 **2** [3] (Figure 1). Additionally, the motif is commonly incorporated into synthetic pharmaceutical candidates, for example in the factor XIa inhibitor **3** [4], the orally-active renin inhibitor **4** [5] and the threonine tyrosine kinase inhibitor CFI-401870 (**5**) [6].

The 2-(1-amidoalkyl)pyridines are almost always synthesised by acylation of the related 2-(1-aminoalkyl)pyridines, which can be prepared by reduction of ketimines derived from 2-acylpyridines [5] or 2-cyanopyridines [6], addition of carbon nucleophiles to aldimines derived from pyridine-2-carboxaldehydes [4,7], or nucleophilic substitution of 2-(1-hydroxyalkyl)pyridines [8]. Alternatively, acyclic precursors bearing the amidoalkyl (or protected aminoalkyl) substituent can be applied in de novo construction of the pyridine ring, exemplified by approaches to the core of the cyclothiazomycins [9–10]. Regardless of the method employed, these are multistep protocols, frequently employing moisture-sensitive organometallic agents or reducing agents. In this paper, we report a new one-pot, two-component synthesis of 2-(1-amidoalkyl)pyridines that arises from a formally ‘umpoled’ coupling of an α-(amidoalkyl) anion equivalent with a pyridyl electrophile.

## Results and Discussion

### Reaction discovery

**Scheme 1:**
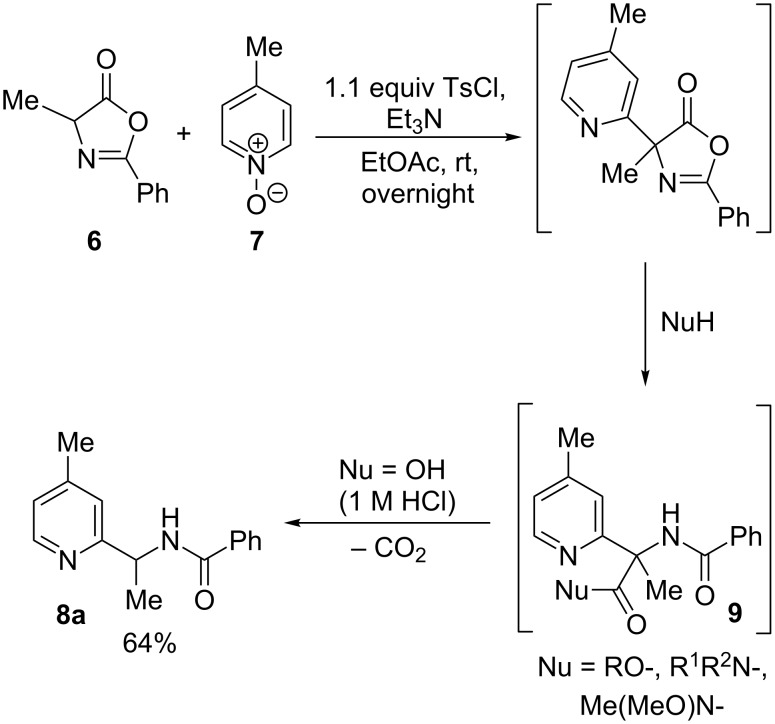
Discovery of the azlactone arylation/decarboxylative hydrolysis approach to 2-(1-amidoalkyl)pyridines.

Our research group has a longstanding interest in the synthesis of α,α-disubstituted amino acids [11–15], and in particular has developed methods for the preparation of α-aryl variants by palladium-catalysed enolate arylation reactions [13–15]. More recently, we have sought to develop more sustainable methods for the arylation of amino acid enolate equivalents that avoid the use of precious metal salts and expensive bespoke ligands, based upon the electrophilic activation of pyridine *N*-oxides and subsequent reaction with acidic carbon nucleophiles [16–20]. Specifically, we have demonstrated that α-pyridyl,α-alkylamino acid derivatives can be prepared in a one-pot three component coupling between readily-available azlactones and pyridine *N*-oxides in the presence of *p*-toluenesulfonyl chloride as an activating agent, followed by opening of the arylated azlactone intermediate with nucleophiles such as alcohols, primary and secondary amines, and *N*,*O*-dialkylhydroxylamines [21] (Scheme 1). However, in the reaction of alanine-derived azlactone **6** with 4-methylpyridine *N*-oxide (**7**) we found that when water (in the form of 1 M HCl) was employed as a nucleophile, the product isolated was in fact the 2-(1-benzamidoethyl)-4-methylpyridine (**8a**), formed by facile decarboxylation of the intermediate pyridylamino acid **9** (Nu = OH) [22–23]. After some minor process optimisation, this product was isolated in 64% yield.

We recognised that this constitutes a formally ‘umpoled’ [24] coupling of an α-amino- or amidoalkyl anion [25–27] with a pyridyl electrophile and hence would complement existing synthetic methods. Given the ready availability of azlactones bearing differential functionality at C2 and C4, a wide range of 2-(1-amidoalkyl)pyridines should become available and we elected to exemplify this process.

### Substrate scope

**Scheme 2:**
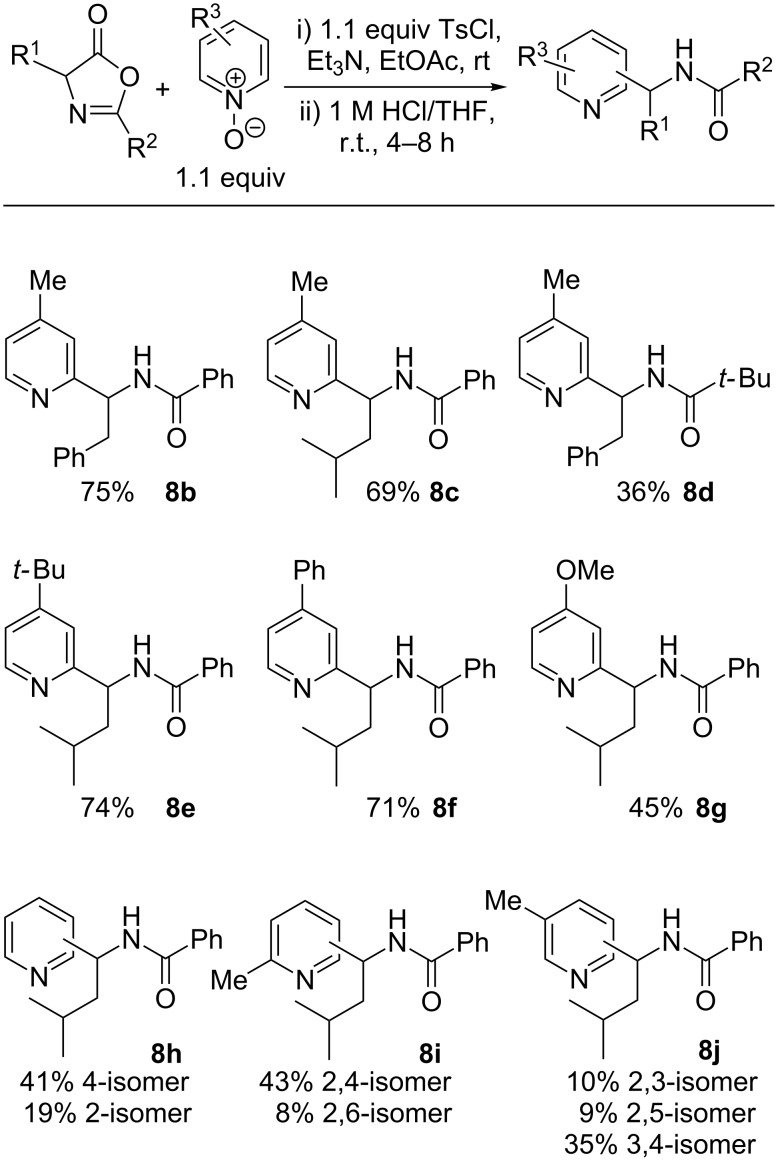
Substrate scope of the direct amidoalkylation of pyridine *N*-oxides.

We first examined variation of azlactone substituents in their reaction with 4-methylpyridine *N*-oxide (Scheme 2). Pleasingly, bulkier C4-substituents such as benzyl or isobutyl groups (derived from phenylalanine and leucine, respectively) were well tolerated, with isolated yields for the products **8b** and **8c** of 75% and 69%, respectively. Variation of the C2-azlactone substituent was also evaluated: swapping a phenyl substituent for a *tert*-butyl substituent gave rise to the pivaloyl amide **8d**, albeit in a modest 36% yield.

Next we examined the behaviour of different 4-substituted pyridine *N*-oxides (either commercially available or prepared by oxidation of the corresponding pyridine with *m*-chloroperbenzoic acid) with a leucine-derived azlactone. The presence of bulkier alkyl or aromatic substituents was well tolerated, giving products **8e** and **8f** in good yield; however, the presence of an electron-donating methoxy group led to a lower yield of **8g** (45%). This may reflect the lower electrophilicity of the activated pyridine *N*-oxide, which may allow non-productive azlactone decomposition pathways to compete with the desired substitution.

Finally, we examined the regiochemical outcome of the reaction of pyridine *N*-oxides with other substitution patterns. Pyridine *N*-oxide itself delivered a ca. 2:1 mixture of the separable 4- and 2-substituted isomers of **8h** in overall 60% yield. Statistically corrected for the available reactive positions, this reflects an inherent 4:1 preference for reaction at the 4-position, which may in part be due to steric hindrance at the C2-positions by the activated *N*-oxide. The high C4-selectivity was also evident in the formation of a >5:1 selectivity for the formation of the 2,4-isomer of **8i** over the 2,6-isomer when 2-methylpyridine *N*-oxide was used as substrate. Finally, the use of 3-methylpyridine *N*-oxide gave a ca. 7:2:2 mixture of 3,4-:2,3-:2,5-isomers of **8j**. Although the regioselectivities are not exceptionally high, the ready chromatographic separation of the various isomers makes this a synthetically tractable approach to 4-substituted (1-amidoalkyl)pyridine derivatives.

## Conclusion

In summary, we have demonstrated a new umpoled disconnection for the one-pot, two-component synthesis of 2-(1-amidoalkyl)pyridines using simple, widely-available coupling partners without the requirement for expensive or critically-available reagents and catalysts. The reactions display good generality over 10 examples (36–75% yields, average 60%). Given the medicinal relevance of the (amidoalkyl)pyridine products, and the convergent nature of the reaction, we believe that the method should find ready application in the concise synthesis of bioactive molecules.

## Supporting Information

File 1 Experimental procedures and full compound characterisation data for products **8a**–**j**.

File 2 Copies of spectra for products **8a**–**j**.
