# Supplementary material for: A convergent, umpoled synthesis of 2-(1-amidoalkyl)pyridines
Source: Beilstein J Org Chem. 2016 Jan 4;12:1–4. doi: 10.3762/bjoc.12.1 (PMC4734400; doi:10.3762/bjoc.12.1)
Supplement: File 1 — Experimental procedures and full compound characterisation data for products 8a–j. [file Beilstein_J_Org_Chem-12-01-s001.pdf]

Supporting Information

for

A convergent, umpoled synthesis of 2-(1-amidoalkyl)pyridines

Tarn C. Johnson and Stephen P. Marsden*

Address: Institute of Process Research and Development, School of Chemistry, University of Leeds, Woodhouse Lane, Leeds LS2 9JT, UK

Email: Stephen P. Marsden* - s.p.marsden@leeds.ac.uk

* Corresponding author

Experimental procedures and full compound characterisation data for products 8a–j

General experimental

All non-aqueous reactions were performed under an atmosphere of nitrogen unless otherwise stated. Water-sensitive reactions were performed in oven-dried glassware, cooled under nitrogen before use. Dichloromethane was dried and purified by means of a Pure Solv MD solvent purification system (Innovative Technology Inc.). Ethyl acetate was dried over 3 Å molecular sieves. All other solvents used were of chromatography or analytical grade. Commercially available starting materials were obtained from Sigma-Aldrich, Fluka, Acros, Alfa-Aesar or Fluorochem and were used without purification.

Thin-layer chromatography (TLC) was carried out on aluminium backed silica plates (Merck silica gel 60 F254). Visualisation of the plates was achieved using an ultraviolet lamp (λ_{max} = 254 nm) and $KMnO_4$. Flash chromatography was carried out using silica gel 60 (60–63 μm particles) supplied by Merck. Strong cation exchange solid-phase extraction (SCX-SPE) was carried out using pre-packed Discovery DSC-SCX cartridges supplied by Supelco.

Infrared spectra were recorded on a Perkin-Elmer Spectrum One FTIR spectrometer with absorption reported in wavenumbers (cm^{-1}). High-resolution mass spectra (HRMS) were recorded on a Bruker MaXis Impact spectrometer with electrospray ionisation (ESI) source. Proton (^1H) and carbon ($^{13}C\{^1H\}$) NMR spectral data were collected on Bruker Advance 500, Bruker DPX500 or DPX300 spectrometers. Chemical shifts (δ) are quoted in parts per million (ppm) and referenced to residual solvent peaks or tetramethylsilane. Coupling constants (*J*) are quoted in Hertz (Hz) and splitting patterns reported in an abbreviated manner: s (singlet), d (doublet), t (triplet), q (quartet), m (multiplet). Assignments were made with the aid of COSY, DEPT-135 and HMQC experiments.

Azlactones were synthesised according to published procedures [1]. Pyridine *N*-oxides were commercially available and used without further purification with the exception of 4-*tert*-butylpyridine *N*-oxide which was made by oxidation of the corresponding pyridine with *m*-chloroperbenzoic acid [2].

Azlactone arylations

General procedure

Triethylamine (0.147 mL, 1.05 mmol) was added to a solution of azlactone (0.50 mmol), 4-toluenesulfonyl chloride (105 mg, 0.55 mmol) and pyridine *N*-oxide (0.55 mmol) in ethyl acetate (2.5 mL) and the mixture was allowed to stir at rt overnight after which the solvent was removed under reduced pressure. Tetrahydrofuran (0.75 mL) was added followed by an aqueous 1 M HCl solution (1.25 mL) and the mixture was stirred until the arylated azlactone was consumed (LCMS). A 2 M NaOH solution (10 mL) was added and the mixture was extracted with ethyl acetate (3 × 10 mL), dried ($MgSO_4$), filtered and the volatiles were removed under reduced pressure.

Compound **8b**

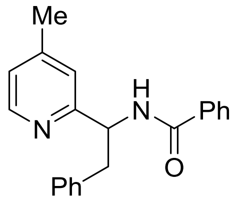

Column chromatography on silica with a gradient elution from 0–60% ethyl acetate in hexane followed by an SCX column gave the product as a colourless, amorphous solid (119 mg, 0.38 mmol, 75%). δ_H **(500 MHz, $CDCl_3$)** 8.37-8.41 (1H, m, 6-Py*H*), 7.80-7.84 (2H, m, Ar*H*), 7.72 (1H, d, *J* 7.5, N*H*), 7.44-7.49 (1H, m, Ar*H*), 7.38-7.43 (2H, m, Ar*H*), 7.13-7.21 (3H, m, Ar*H*), 6.96-7.01 (3H, m, 5-Py*H*, Ar*H*), 6.74-6.77 (1H, m, 3-Py*H*), 5.40-5.47 (1H, m, HNC*H*), 3.36 (1H, dd, *J* 13.3, 5.6, C*H*H), 3.18 (1H, dd, *J* 13.3, 7.9, CH*H*), 2.23 (PyCH_3); δ_C **(125 MHz, $CDCl_3$)** 166.4 (Q), 158.4 (Q), 148.5, 147.6 (Q), 137.2 (Q), 134.6 (Q), 131.3, 129.5, 128.4, 128.0, 126.9, 126.3, 123.5, 123.4, 55.4, 42.3 (CH_2), 20.8; **(Found (ESI):** M^+ + H, 317.1649. $C_{21}H_{21}N_2O$ requires 317.1648); ν_{max} 3301, 1627, 1542, 1492, 1304, 821, 698, 669, 499, 447 cm^{-1}.

Compound **8c**

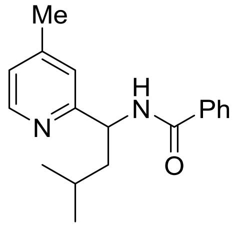

Column chromatography on silica with a gradient elution from 0–60% ethyl acetate in hexane gave the product as a yellow, crystalline solid (98 mg, 0.35 mmol, 69%). **Mp** 100-103 °C ($CHCl_3$); δ_H **(500 MHz, $CDCl_3$)** 8.36-8.40 (1H, m, 6-Py*H*), 7.78-7.84 (2H, m, Ar*H*), 7.40-7.46 (1H, m, Ar*H*), 7.34-7.40 (3H, m, N*H*, Ar*H*), 7.08-7.11 (1H, m, 3-Py*H*), 6.94-6.99 (1H, m, 5-Py*H*), 5.26-5.33 (1H, m, HNC*H*), 2.31 (3H, s, PyCH_3), 1.76-1.84 (1H, m, C*H*H), 1.66-1.74 (1H, m, CH*H*), 1.54-1.64 (1H, m, (C*H*(CH_3)$_2$)), 0.91-0.99 (6H, m, (CH(CH_3)$_2$)); δ_C **(125 MHz, $CDCl_3$)** 166.4 (Q), 160.5 (Q), 149.0, 147.7 (Q), 134.6 (Q), 131.2, 128.3, 126.9, 123.2, 123.0, 52.6, 45.9 (CH_2), 24.9, 22.8, 22.6, 20.9**; (Found (ESI):** M^+ + H, 283.1806. $C_{18}H_{23}N_2O$ requires 283.1805); ν_{max} 3317, 2923, 1630, 1602, 1539, 1490, 1298, 697, 663, 486 cm^{-1}.

Compound **8d**

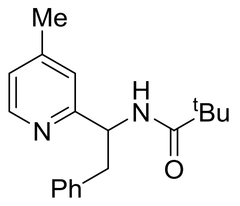

Column chromatography on silica with a gradient elution from 0–60% ethyl acetate in hexane followed by an SCX column gave the product as a colourless, crystalline solid (54 mg, 0.18 mmol, 36%). **Mp** 100-103 °C ($CHCl_3$); **δ_H (500 MHz, $CDCl_3$)** 8.35-8.39 (1H, m, 6-Py*H*), 7.11-7.19 (3H, m, Ar*H*), 7.04 (1H, d, *J* 7.1, N*H*), 6.91-6.97 (3H, m, 5-Py*H*, Ar*H*), 6.65-6.68 (1H, m, 3-Py*H*), 5.14-5.20 (1H, m, HNC*H*), 3.20 (3H, dd, *J* 13.2, 5.8, C*H*H), 3.02 (1H, dd, *J* 13.2, 7.9, CH*H*), 2.21 (3H, s, PyC*H*$_3$), 1.16 (9H, s, C(C*H*$_3$)$_3$); **δ_C (125 MHz, $CDCl_3$)** 177.6 (Q), 158.8 (Q), 148.7, 147.3 (Q), 137.4 (Q), 129.5, 128.0, 126.3, 123.5, 123.3, 54.9, 42.3 (CH_2), 38.6 (Q), 27.4, 20.8; **(Found (ESI):** M^+ + H, 297.1967. $C_{19}H_{25}N_2O$ requires 297.1961); ν_{max} 3330, 2959, 1634, 1601, 1530, 1210, 840, 755, 697, 669, 494 cm^{-1}.

Compound **8e**

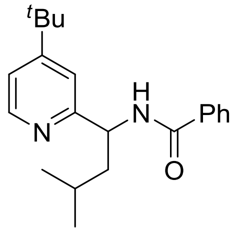

Column chromatography on silica with a gradient elution from 0–60% ethyl acetate in hexane followed by an SCX column gave the product as a colourless, crystalline solid (121 mg, 0.37 mmol, 74%). **Mp** 109-110 °C ($CHCl_3$); **δ_H (500 MHz, $CDCl_3$)** 8.45-8.48 (1H, m, 6-Py*H*), 7.82-7.87 (2H, m, Ar*H*), 7.38-7.49 (4H, m, N*H*, Ar*H*), 7.27-7.29 (1H, m, 3-Py*H*), 7.17-7.21 (1H, m, 5-Py*H*), 5.34-5.41 (1H, m, HNC*H*), 1.82-1.89 (1H, m, C*H*H), 1.66-1.73 (1H, m, CH*H*), 1.57-1.65 (1H, m, (C*H*(CH_3)$_2$)), 1.31 (9H, s, C(C*H*$_3$)$_3$), 0.95-1.02 (6H, m, (CH(C*H*$_3$)$_2$)); **δ_C (125 MHz, $CDCl_3$)** 166.4 (Q), 161.1 (Q), 160.4 (Q), 148.9, 134.6 (Q), 131.1, 128.3, 126.9, 119.4, 119.2, 52.7, 46.1 (CH_2), 34.6 (Q), 30.4, 24.9, 22.7, 22.5; **(Found (ESI):** M^+ + H, 325.2279. $C_{21}H_{29}N_2O$ requires 325.2274); ν_{max} 3326, 2958, 1638, 1600, 1522, 1488, 728, 694, 533 cm^{-1}.

Compound **8f**

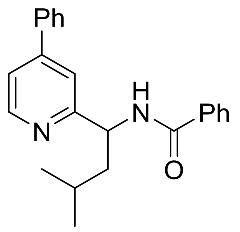

Column chromatography on silica with a gradient elution from 0–80% ethyl acetate in hexane followed by an SCX column gave the product as a yellow, crystalline solid (122 mg, 0.35 mmol, 71%). **Mp** 104-106 °C (EtOAc/pentane); δ_H **(500 MHz, $CDCl_3$)** 8.58-8.62 (1H, m, 6-Py*H*), 7.83-7.88 (2H, m, Ar*H*), 7.61-7.65 (2H, m, Ar*H*), 7.51-7.54 (1H, m, 3-Py*H*), 7.37-7.49 (8H, m, N*H*, Ar*H*, 5-Py*H*), 5.43-5.51 (1H, m, HNC*H*), 1.85-1.93 (1H, m, C*H*H), 1.75-1.82 (1H, m, CH*H*), 1.62-1.71 (1H, m, ($CH(CH_3)_2$)), 0.97-1.04 (6H, m, ($CH(CH_3)_2$)); δ_C **(125 MHz, $CDCl_3$)** 166.5 (Q), 161.3 (Q), 149.7, 149.1 (Q), 137.9 (Q), 134.6 (Q), 131.2, 129.0, 128.9, 128.3, 127.0, 120.3, 120.1, 52.8, 46.0 (CH_2), 25.0, 22.8, 22.6, one ArC not found; **(Found (ESI):** M^+ + H, 345.1969. $C_{23}H_{25}N_2O$ requires 345.1961); ν_{max} 3291, 2968, 1634, 1595, 1534, 1317, 1292, 754, 724, 670, 658 cm^{-1}.

Compound **8g**

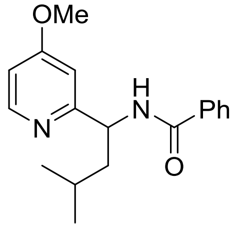

Column chromatography on silica with a gradient elution from 0–80% ethyl acetate in hexane gave the product as a colourless, crystalline solid (67 mg, 0.23 mmol, 45%). **Mp** 119-120 °C ($CHCl_3$); δ_H **(500 MHz, $CDCl_3$)** 8.32-8.37 (1H, m, 6-Py*H*), 7.78-7.83 (2H, m, Ar*H*), 7.41-7.45 (1H, m, Ar*H*), 7.34-7.40 (3H, m, N*H*, Ar*H*), 6.79-6.82 (1H, m, 3-Py*H*), 6.66-6.69 (1H, m, 5-Py*H*), 5.24-5.30 (1H, m, HNC*H*), 3.80 (3H, s, OCH_3), 1.77-1.84 (1H, m, C*H*H), 1.67-1.74 (1H, m, CH*H*), 1.55-1.64 (1H, m, ($CH(CH_3)_2$)), 0.92-0.98 (6H, m, ($CH(CH_3)_2$)); δ_C **(125 MHz, $CDCl_3$)** 166.5 (Q), 166.0 (Q), 162.5 (Q), 150.5, 134.6 (Q), 131.2, 128.3, 127.0, 108.5, 108.1, 55.1, 52.8, 45.7 (CH_2), 24.9, 22.7, 22.6; **(Found (ESI):** M^+ + H, 299.1760. $C_{18}H_{23}N_2O_2$ requires 299.1754); ν_{max} 3328, 2945, 1633, 1593, 1539, 1482, 1077, 701, 670 cm^{-1}.

Compound **8h**

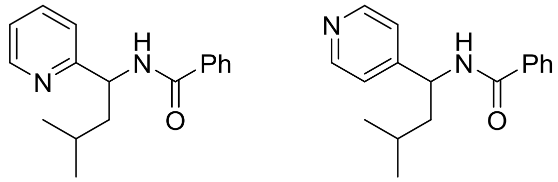

Column chromatography on silica with a gradient elution from 0–100% ethyl acetate in hexane gave the 2-regioisomer as a colourless oil and the 4-regioisomer as a colourless, amorphous solid (55 mg, 0.21 mmol, 41%). The 2-regioisomer was further purified by SCX column to give a colourless, crystalline solid (25 mg, 0.09 mmol, 19%). **2-Regioisomer; Mp** 109-110 °C (EtOAc/pentane); **δ_H (500 MHz, $CDCl_3$)** 8.53-8.58 (1H, m, 6-Py*H*), 7.80-7.85 (2H, m, Ar*H*), 7.61-7.67 (1H, m, 4-Py*H*), 7.44-7.49 (1H, m, Ar*H*), 7.38-7.44 (2H, m, Ar*H*), 7.27-7.37 (2H, m, N*H*, Ar*H*), 7.15-7.20 (1H, m, 5-Py*H*), 5.33-5.39 (1H, m, HNC*H*), 1.78-1.86 (1H, m, C*H*H), 1.69-1.76 (1H, m, CH*H*), 1.55-1.64 (1H, m, (C*H*(CH_3)$_2$), 0.92-1.01 (6H, m, (CH(CH_3)$_2$); **δ_C (125 MHz, $CDCl_3$)** 166.5 (Q), 160.8 (Q), 149.4, 136.6, 134.7 (Q), 131.3, 128.4, 127.0, 122.3, 122.2, 52.7, 46.1 (CH_2), 25.0, 22.8, 22.7; **(Found (ESI):** M^+ + H, 269.1653. $C_{17}H_{21}N_2O$ requires 269.1648); ν_{max} 3372, 3045, 2936, 1645, 1589, 1514, 1485, 1469, 1434, 1311, 767, 751, 718, 691, 548 cm^{-1}. **4-Regioisomer; δ_H (500 MHz, $CDCl_3$)** 8.40-8.50 (2H, m, 2,6-Py*H*), 7.72-7.79 (2H, m, Ar*H*), 7.41-7.48 (1H, m, Ar*H*), 7.30-7.37 (2H, m, Ar*H*), 7.14-7.26 (3H, m, N*H*, 3,5-Py*H*), 5.14-5.21 (1H, m, HNC*H*), 1.68-1.78 (1H, m, C*H*H), 1.55-1.67 (2H, m, CH*H*, (C*H*(CH_3)$_2$), 0.86-0.97 (6H, m, (CH(CH_3)$_2$); **δ_C (125 MHz, $CDCl_3$)** 167.2 (Q), 152.1 (Q), 149.8, 134.1 (Q), 131.6, 128.5, 127.0, 121.5, 51.2, 44.9 (CH_2), 25.0, 22.8, 22.0; **(Found (ESI):** M^+ + H, 269.1654. $C_{17}H_{21}N_2O$ requires 269.1648); ν_{max} 3277, 3051, 2956, 1632, 1600, 1530, 1489, 1412, 1230, 1217, 823, 803, 694, 563 cm^{-1}.

Compound **8i**

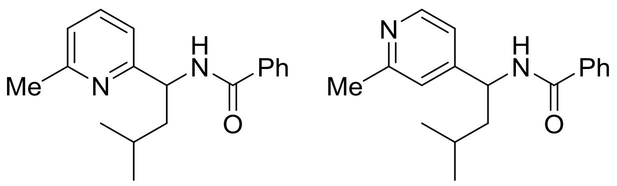

Column chromatography on silica with a gradient elution from 0–100% ethyl acetate in hexane followed by 10% methanol in ethyl acetate gave the 2,6-regioisomer as a colourless oil and the 2,4-regioisomer as a yellow, crystalline solid (61 mg, 0.22 mmol, 43%). The 2,6-regioisomer was further

purified by SCX column and mass-directed preparative HPLC with a gradient elution from 5–95% methanol in water to give a colourless, crystalline solid (12 mg, 0.04 mmol, 8%). **2,6-Regioisomer; Mp** 136-137 °C (EtOAc/pentane); **δ_H (500 MHz, $CDCl_3$)** 7.85-7.91 (2H, m, Ar*H*), 7.54-7.66 (1H, m, Py*H*), 7.46-7.51 (1H, m, Ar*H*), 7.41-7.46 (2H, m, Ar*H*), 7.11-7.17 (1H, m, Py*H*), 7.06-7.11 (1H, m, Py*H*), 5.29-5.38 (1H, m, HNC*H*), 2.60 (3H, s, PyCH_3), 1.82-1.94 (1H, m, C*H*H), 1.67-1.74 (1H, m, CH*H*), 1.57-1.66 (1H, m, (C*H*$(CH_3)_2$), 0.98 (6H, d, *J* 6.4, (CH$(CH_3)_2$), N*H* not found; **δ_C (125 MHz, $CDCl_3$)** 166.6 (Q), 159.7 (Q), 157.9 (Q), 137.5, 134.6 (Q), 131.3, 128.5, 127.1, 122.2, 119.5, 52.4, 46.0 (CH_2), 25.0, 22.8, 22.6; **(Found (ESI):** M^+ + H, 283.1811. $C_{18}H_{23}N_2O$ requires 283.1805); **ν_{max}** 3281, 3063, 2956, 1713, 1577, 1493, 1367, 1334, 787, 695 cm^{-1}. **2,4-Regioisomer; Mp** 97-99 °C ($CHCl_3$); **δ_H (500 MHz, $CDCl_3$)** 8.33-8.38 (1H, m, 2-Py*H*), 7.73-7.78 (2H, m, Ar*H*), 7.42-7.48 (1H, m, Ar*H*), 7.32-7.38 (2H, m, Ar*H*), 7.06-7.10 (1H, m, 5-Py*H*), 7.00-7.05 (1H, m, 3-Py*H*), 6.86-6.92 (1H, d, *J* 7.9, N*H*), 5.11-5.18 (1H, m, HNC*H*), 2.46 (3H, s, Py(CH_3), 1.68-1.77 (1H, m, C*H*H), 1.57-1.66 (2H, m, CH*H*, (C*H*$(CH_3)_2$), 0.88-0.97 (6H, m, (CH$(CH_3)_2$); **δ_C (125 MHz, $CDCl_3$)** 167.0 (Q), 158.6 (Q), 152.3 (Q), 149.2, 134.1 (Q), 131.6, 128.5, 126.9, 121.2, 118.5, 51.3, 45.0 (CH_2), 25.1, 24.3, 22.8, 22.0**; (Found (ESI):** M^+ + H, 283.1811. $C_{18}H_{23}N_2O$ requires 283.1805); **ν_{max}** 3246, 2956, 1634, 1603, 1532, 1489, 1290, 751, 690, 602 cm^{-1}.

Compound **8j**

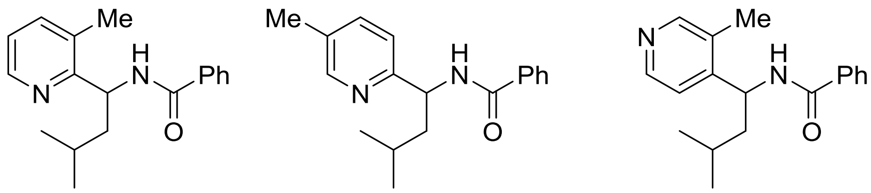

Column chromatography on silica with a gradient elution from 0–100% ethyl acetate in hexane gave the 2,3, 2,5 and 3,4-regioisomers. The 2,3-regioisomer was further purified by SCX column to give a colourless film (14 mg, 0.05 mmol, 10%). The 2,5-regioisomer was further purified by SCX column and mass-directed HPLC with a gradient elution from 5–95% methanol in water to give a colourless film (13 mg, 0.05 mmol, 9%). The 3,4-regioisomer was further purified by SCX column to give a colourless, crystalline solid (49 mg, 0.17 mmol, 35%). **2,3-Regioisomer; δ_H (500 MHz, $CDCl_3$)** 8.37-8.41 (1H, m, 6-Py*H*), 7.85-7.89 (2H, m, Ar*H*), 7.64-7.71 (1H, m, N*H*), 7.45-7.52 (2H, m, 4-Py*H*, Ar*H*), 7.40-7.45 (2H, m, Ar*H*), 7.10-7.14 (1H, m, 5-Py*H*), 5.62-5.68 (1H, m, HNC*H*), 2.47 (3H, s, PyCH_3),

1.72-1.84 (2H, m, C*H*H, (C*H*(CH_3)$_2$)), 1.51-1.59 (1H, m, CH*H*), 1.08 (3H, d, *J* 6.5 (CH_3)CH(CH_3)), 0.94 (3H, d, *J* 6.5, (CH_3)CH(CH_3)); **δ_C (125 MHz, $CDCl_3$)** 166.7 (Q), 159.2 (Q), 146.0, 138.8, 134.6 (Q), 131.3, 130.3 (Q), 128.4, 127.1, 122.3, 48.4, 45.9 (CH_2), 25.1, 23.5, 22.1, 18.1; **(Found (ESI):** M^+ + H, 283.1811. $C_{18}H_{23}N_2O$ requires 283.1805); **ν_{max}** 3342, 2954, 1644, 1577, 1468, 1450, 1384, 1350, 1289, 790, 709, 692, 590 cm^{-1}. **2,5-Regioisomer; δ_H (500 MHz, $CDCl_3$)** 8.38-8.41 (1H, m, 6-Py*H*), 7.83-7.87 (2H, m, Ar*H*), 7.39-7.55 (5H, m, N*H*, Ar*H*, 4-Py*H*), 7.24-7.28 (1H, m, 3-Py*H*), 5.31-5.37 (1H, m, HNC*H*), 2.34 (3H, s, PyCH_3), 1.84-1.91 (1H, m, C*H*H), 1.70-1.77 (1H, m, CH*H*), 1.53-1.64 (1H, m, (C*H*(CH_3)$_2$)), 0.93-1.01 (6H, m, (CH(CH_3)$_2$)); **δ_C (125 MHz, $CDCl_3$)** 166.6 (Q), 157.6 (Q), 148.9, 138.0, 134.5 (Q), 132.2 (Q), 131.3, 128.5, 127.1, 122.2, 52.2, 45.8 (CH_2), 25.0, 22.7, 22.6, 18.1; **(Found (ESI):** M^+ + H, 283.1807. $C_{18}H_{23}N_2O$ requires 283.1805); **ν_{max}** 3324, 2954, 1631, 1487, 1467, 1288, 693, 545 cm^{-1}. **3,4-Regioisomer; Mp** 159-160 °C ($CHCl_3$); **δ_H (500 MHz, $CDCl_3$)** 8.30 (2H, m, 2,6-Py*H*), 7.73-7.78 (2H, m, Ar*H*), 7.41-7.47 (1H, m, Ar*H*), 7.31-7.37 (2H, m, Ar*H*), 7.17-7.21 (1H, m, 5-Py*H*), 7.14 (1H, d, *J* 7.7, N*H*), 5.34-5.40 (1H, m, HNC*H*), 2.40 (3H, s, PyCH_3), 1.66-1.76 (2H, m, C*H*H, (C*H*(CH_3)$_2$)), 1.47-1.54 (1H, m, CH*H*), 0.98 (3H, d, *J* 6.4, (CH_3)CH(CH_3)), 0.92 (3H, d, *J* 6.4, (CH_3)CH(CH_3)); **δ_C (125 MHz, $CDCl_3$)** 167.1 (Q), 151.2 (Q), 150.5, 147.2, 134.0 (Q), 131.5, 130.9 (Q), 128.4, 127.0, 119.9, 48.0, 44.4 (CH_2), 25.3, 23.1, 21.7, 16.0; **(Found (ESI):** M^+ + H, 283.1807. $C_{18}H_{23}N_2O$ requires 283.1805); **ν_{max}** 3283, 2955, 1636, 1536, 1314, 688, 579 cm^{-1}.

References

1. Macovei, C.; Vicenatti, P.; Quinton, J.; Nevers, M-C.; Volland, H.; Créminon, C; Taran, F. *Chem. Commun.*, **2012**, *48*, 4411-4413.

2. Kokatla, H.P.; Thomson, P.F.; Bae, S.; Doddi, V.R.; Lakshman, M.K. *J. Org. Chem.*, **2011**, *76*, 7842-7848.
