# Supplementary material for: A convergent, umpoled synthesis of 2-(1-amidoalkyl)pyridines
Source: Beilstein J Org Chem. 2016 Jan 4;12:1–4. doi: 10.3762/bjoc.12.1 (PMC4734400; doi:10.3762/bjoc.12.1)

**Supporting Information**

**for**

# A convergent, umpoled synthesis of 2-(1-amidoalkyl)pyridines

Tarn C. Johnson and Stephen P. Marsden*

Address: Institute of Process Research and Development, School of Chemistry, University of Leeds, Woodhouse Lane, Leeds LS2 9JT, UK

Email: Stephen P. Marsden* - s.p.marsden@leeds.ac.uk

* Corresponding author

# Copies of spectra for products 8a–j

500 MHz $^{1}$H NMR of **8a** in $CDCl_3$

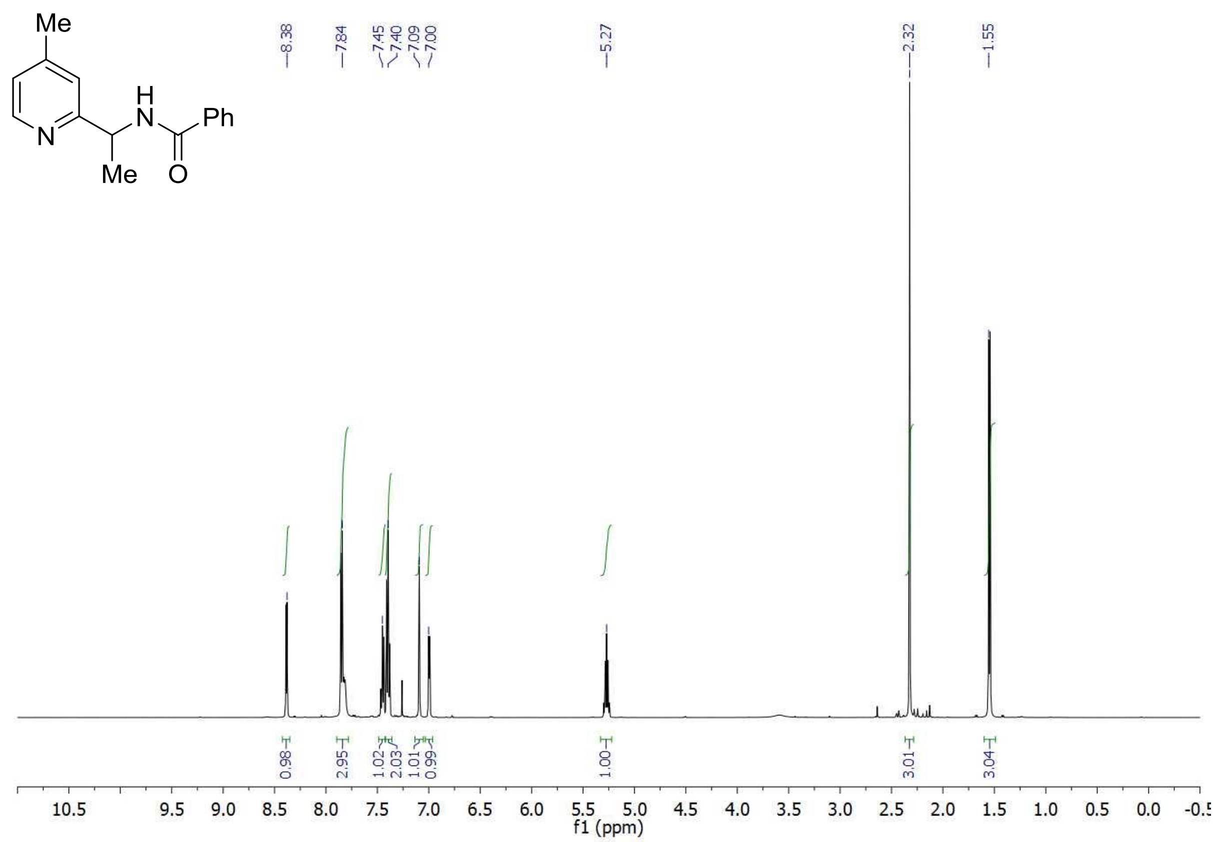

125 MHz $^{13}$C NMR of **8a** in $CDCl_3$

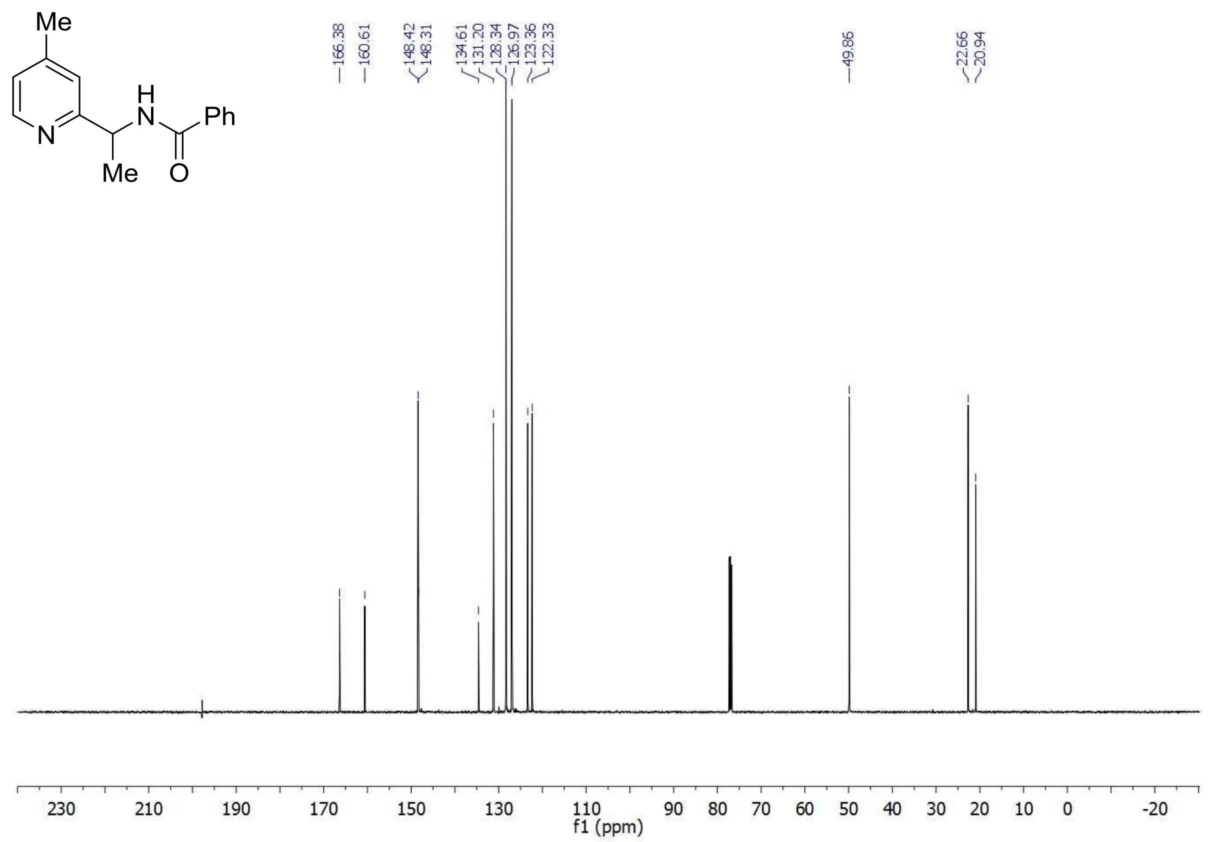

500 MHz $^{1}H$ NMR of **8b** in $CDCl_3$

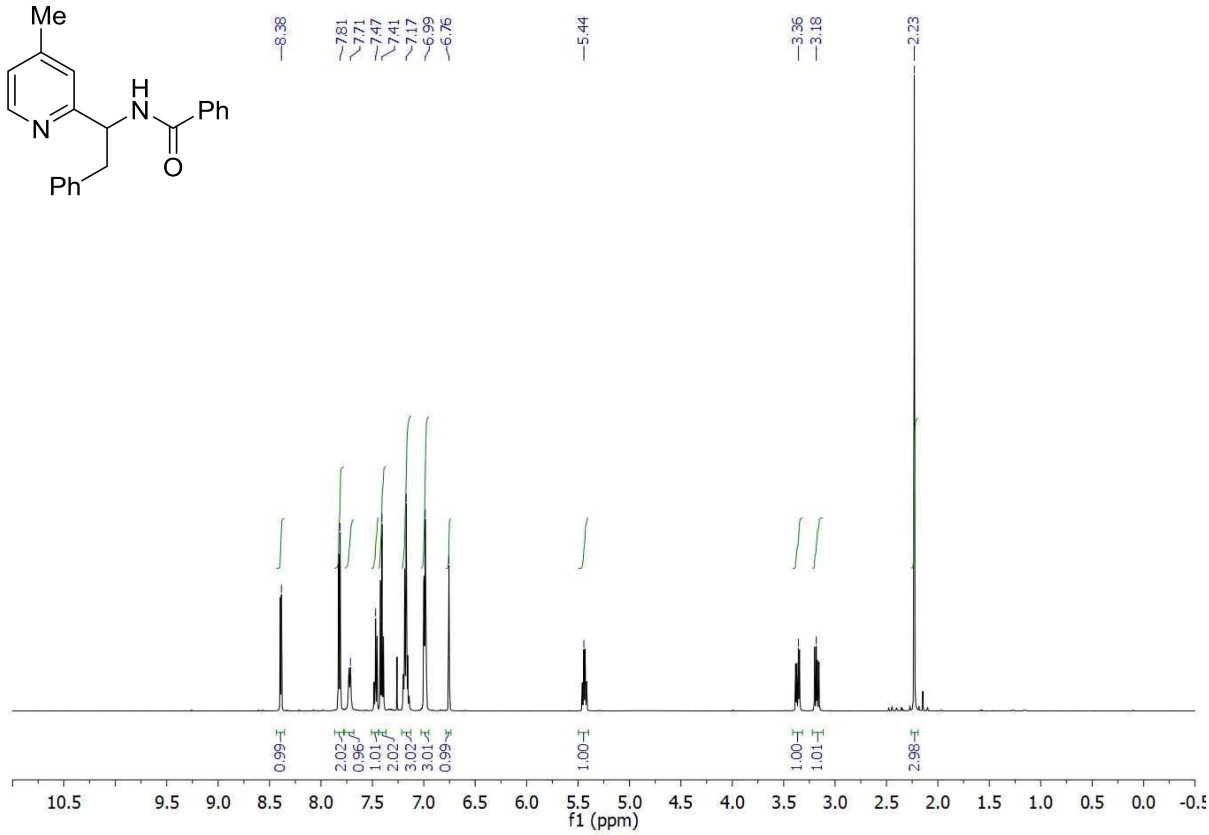


125 MHz $^{13}C$ NMR of **8b** in $CDCl_3$

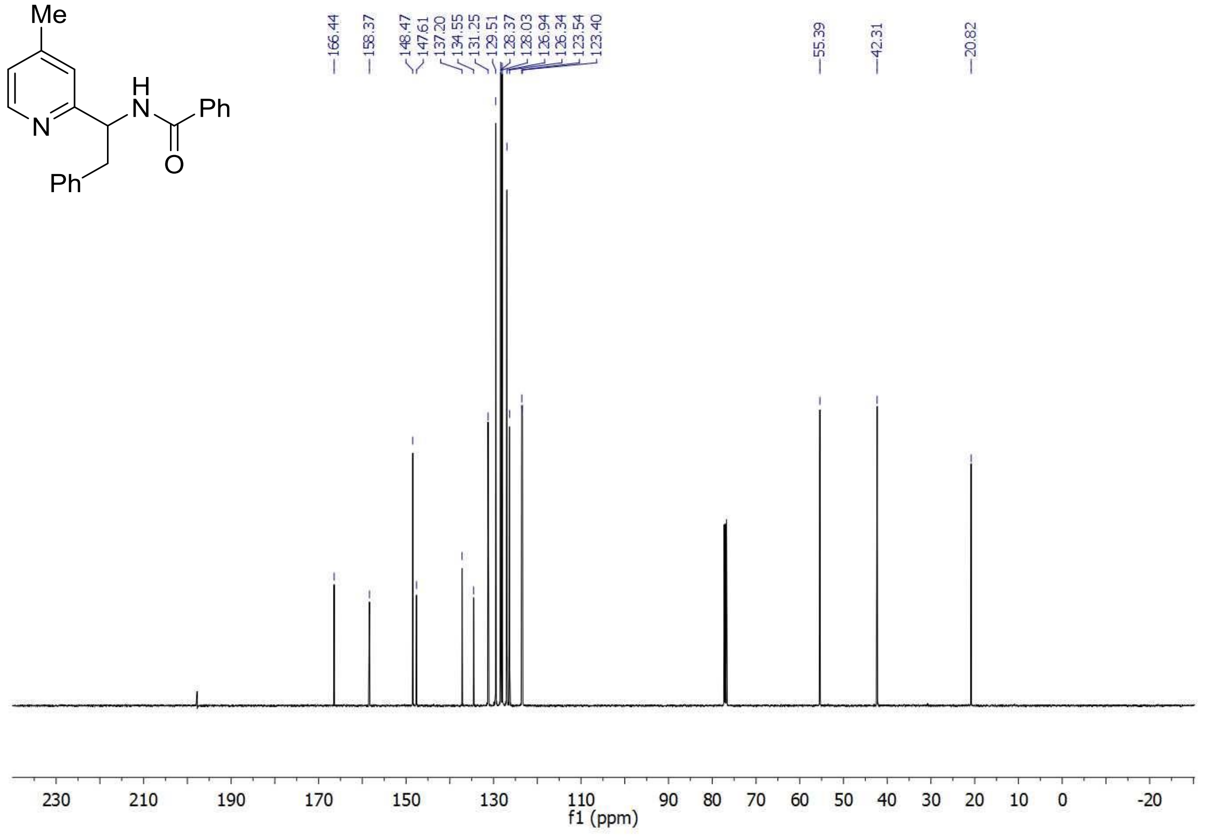

500 MHz $^{1}H$ NMR of **8c** in $CDCl_3$

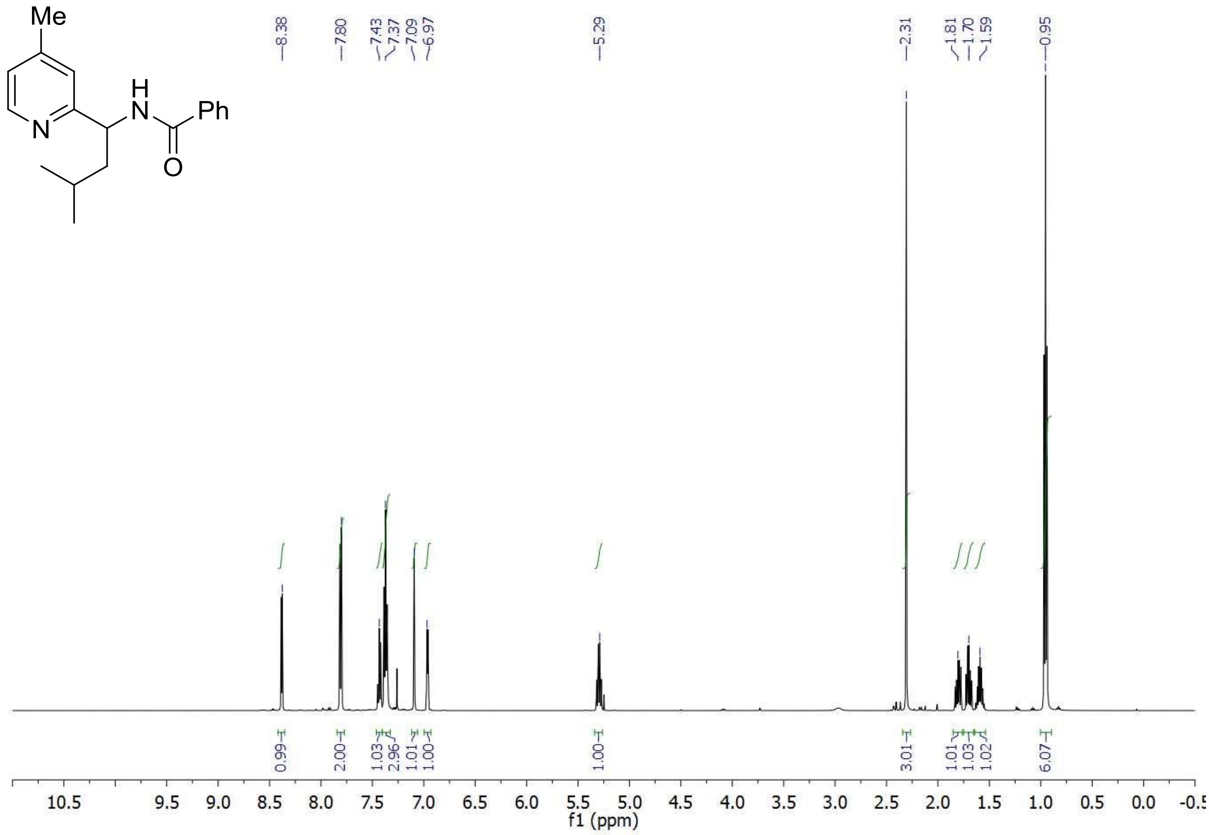


125 MHz $^{13}C$ NMR of **8c** in $CDCl_3$

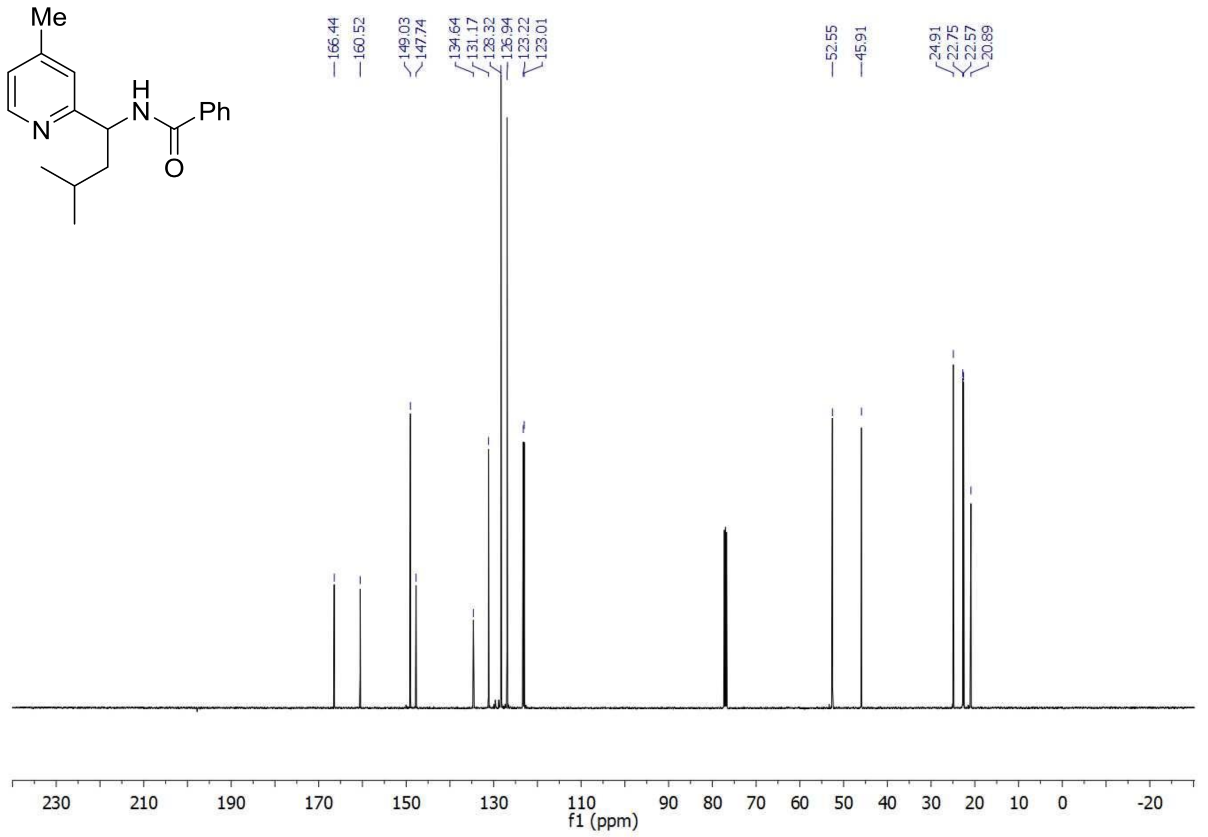

500 MHz $^1H$ NMR of **8d** in $CDCl_3$

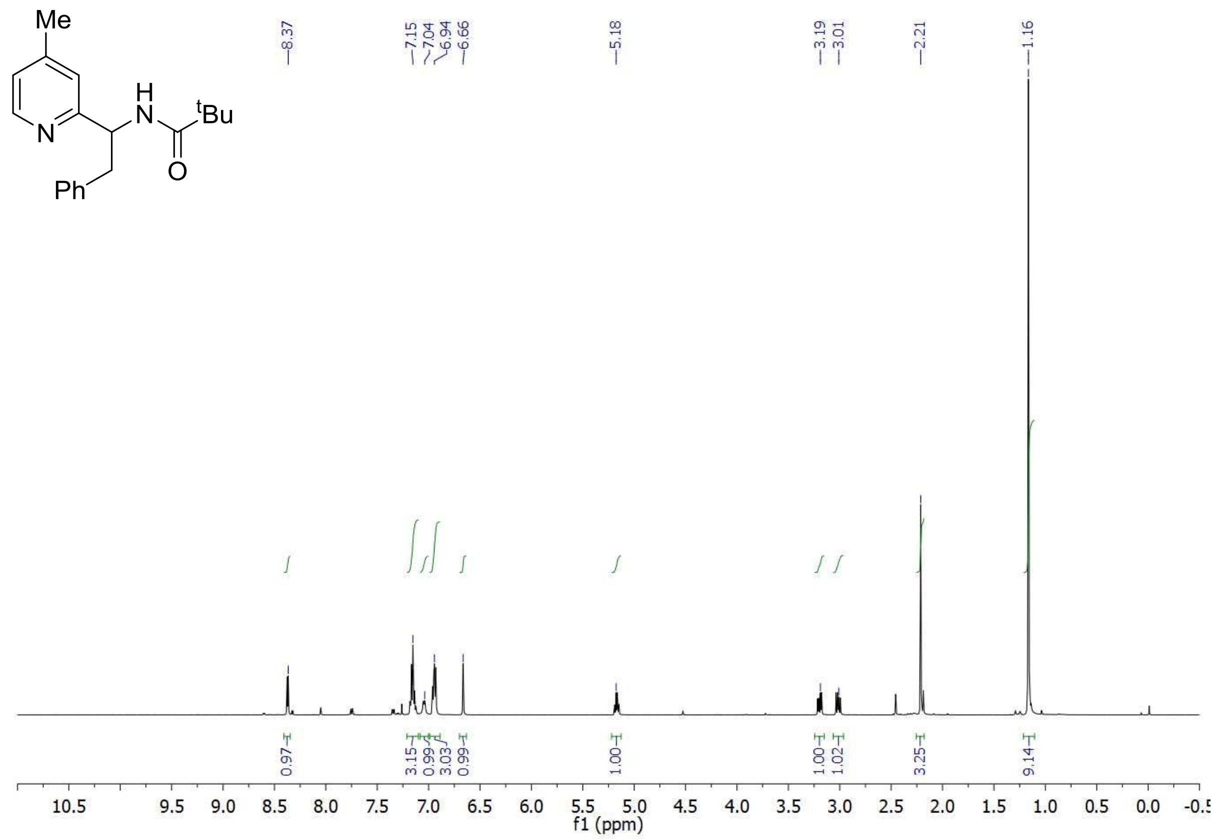


125 MHz $^{13}C$ NMR of **8d** in $CDCl_3$

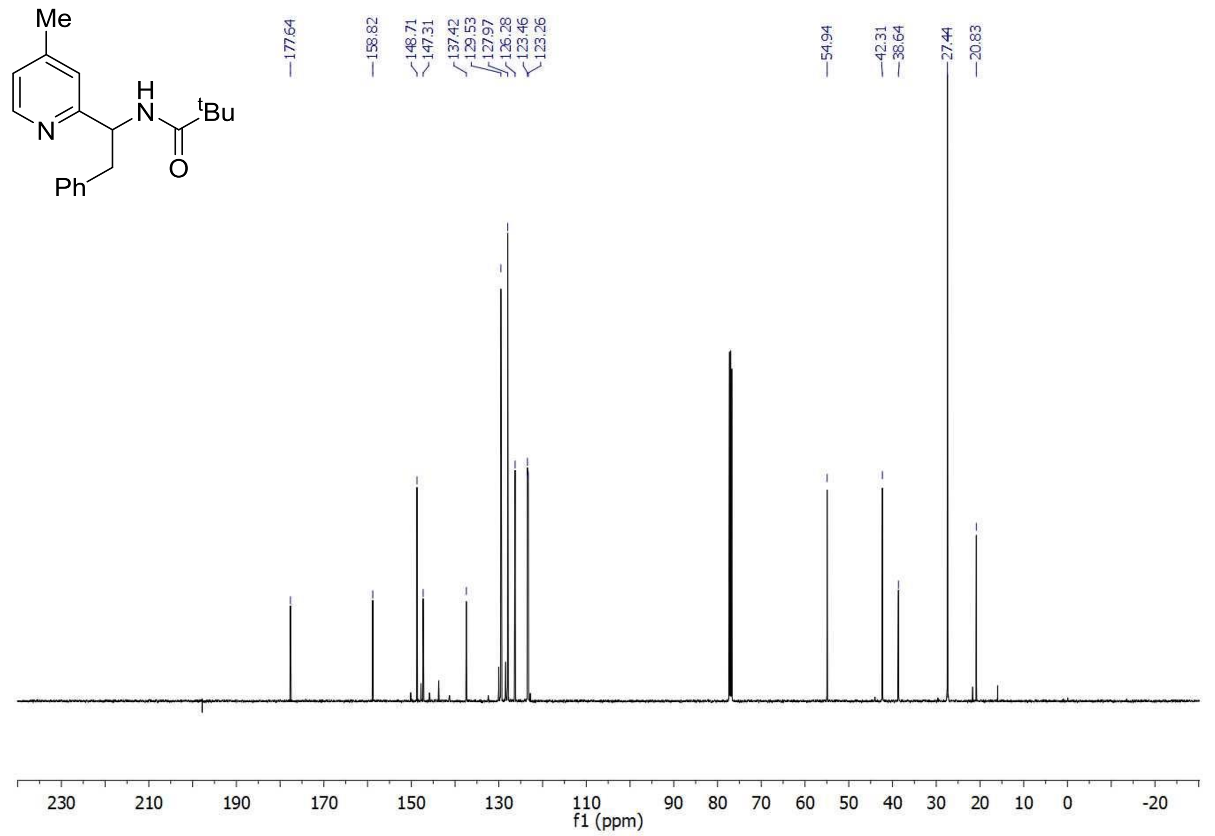

500 MHz $^{1}H$ NMR of **8e** in $CDCl_3$

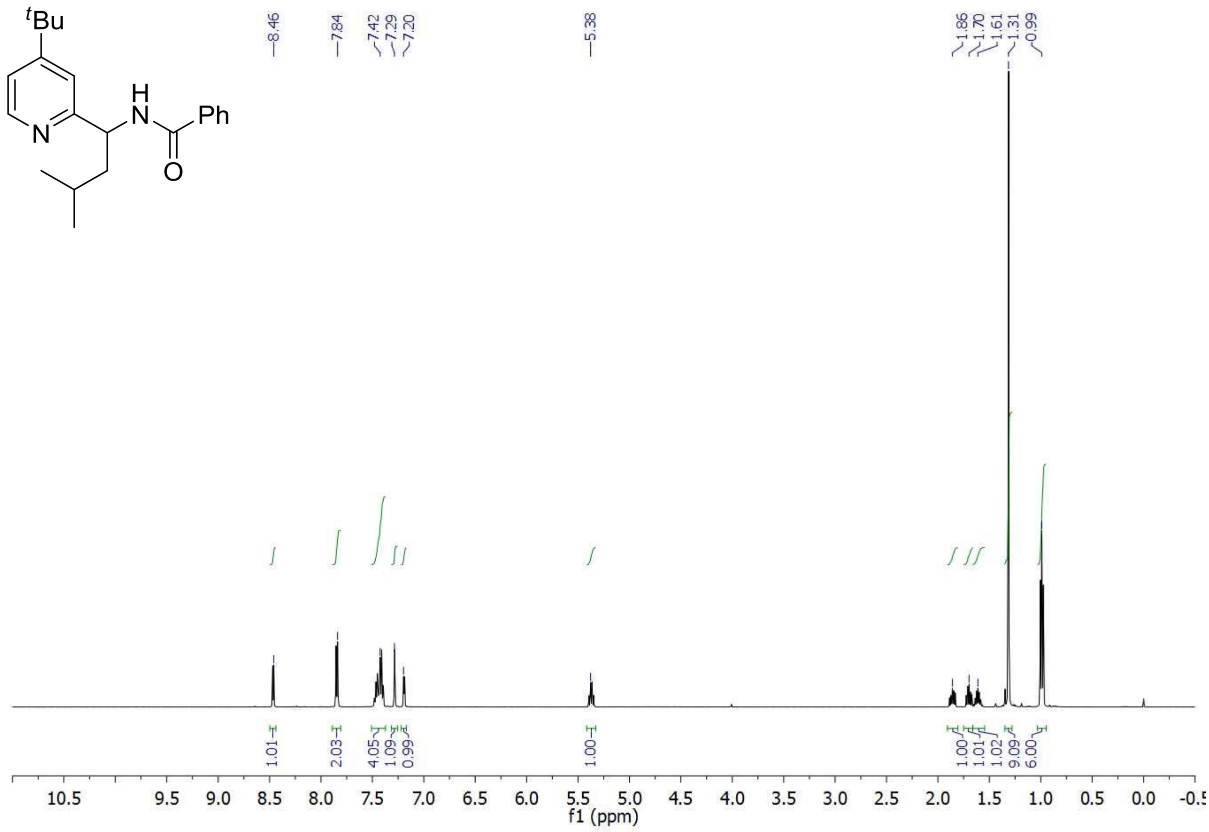


125 MHz $^{13}C$ NMR of **8e** in $CDCl_3$

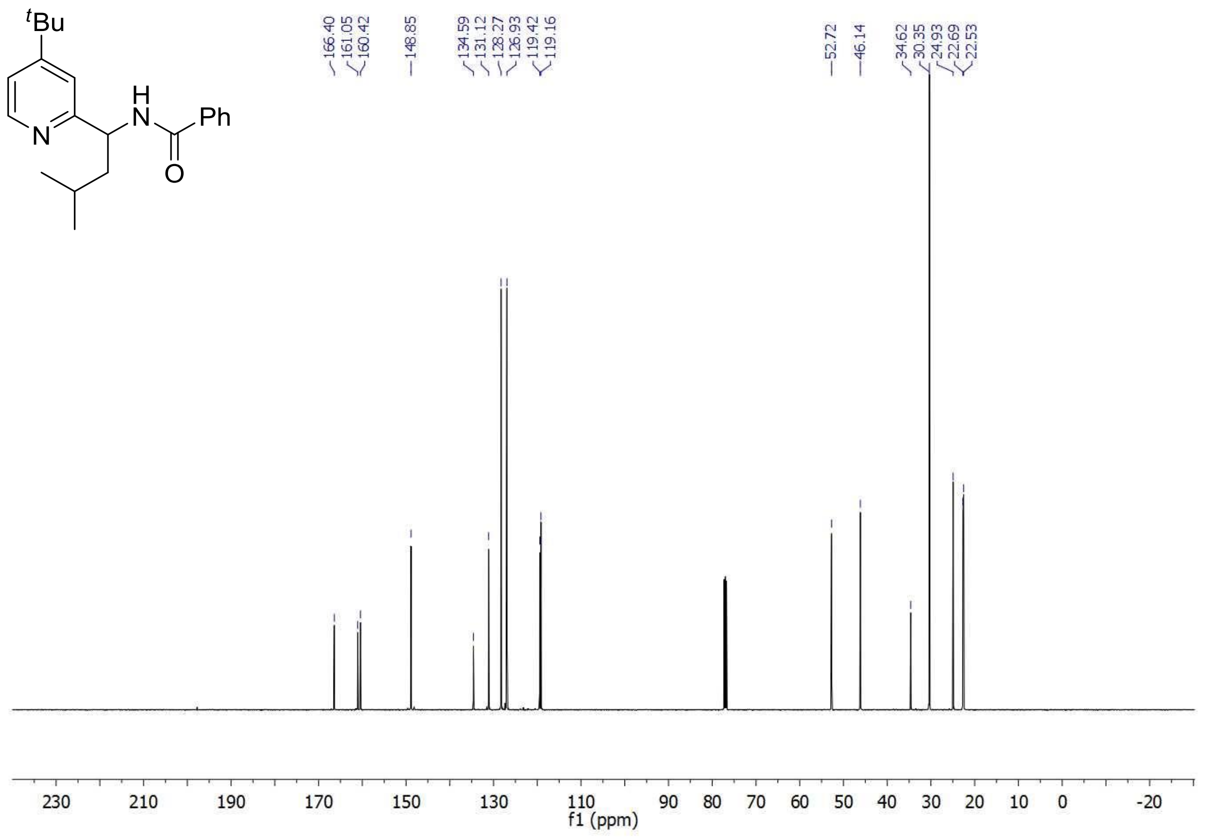

500 MHz $^{1}H$ NMR of **8f** in $CDCl_3$

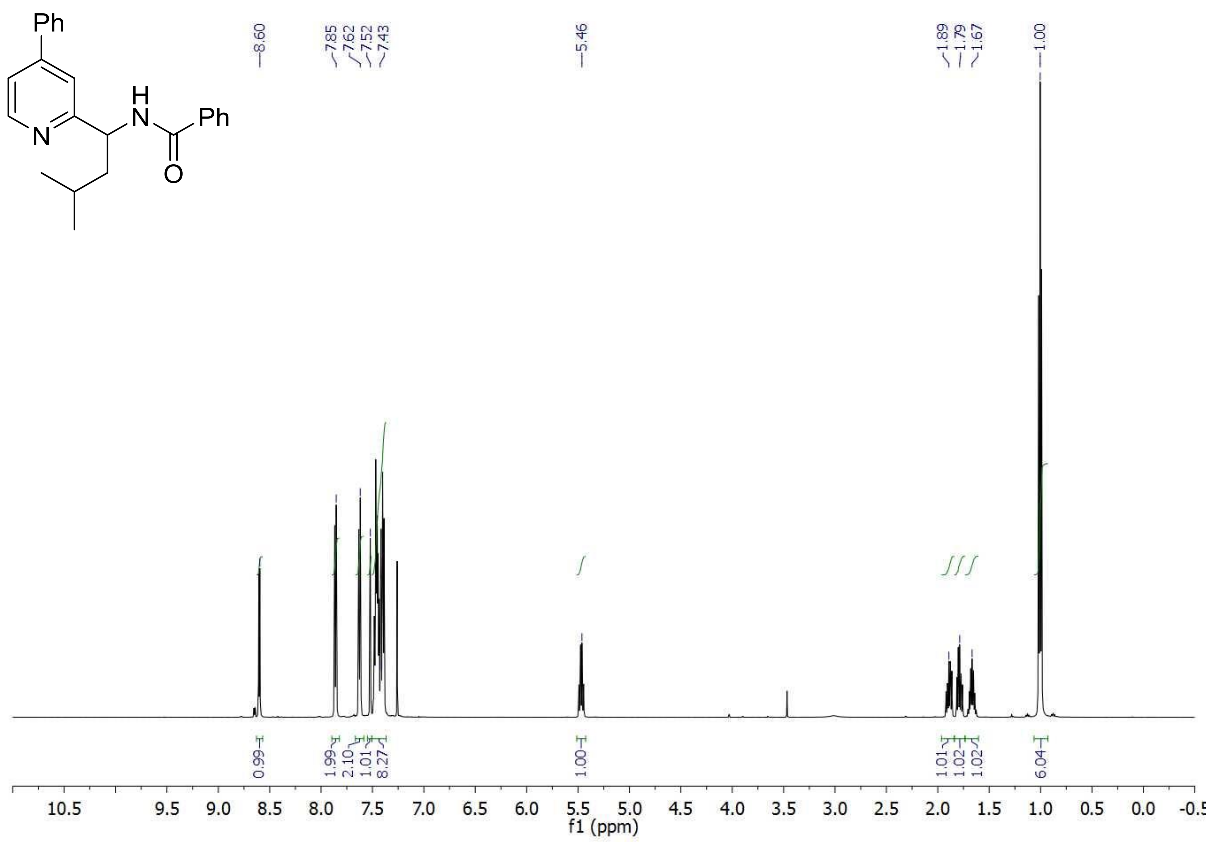


125 MHz $^{13}C$ NMR of **8f** in $CDCl_3$

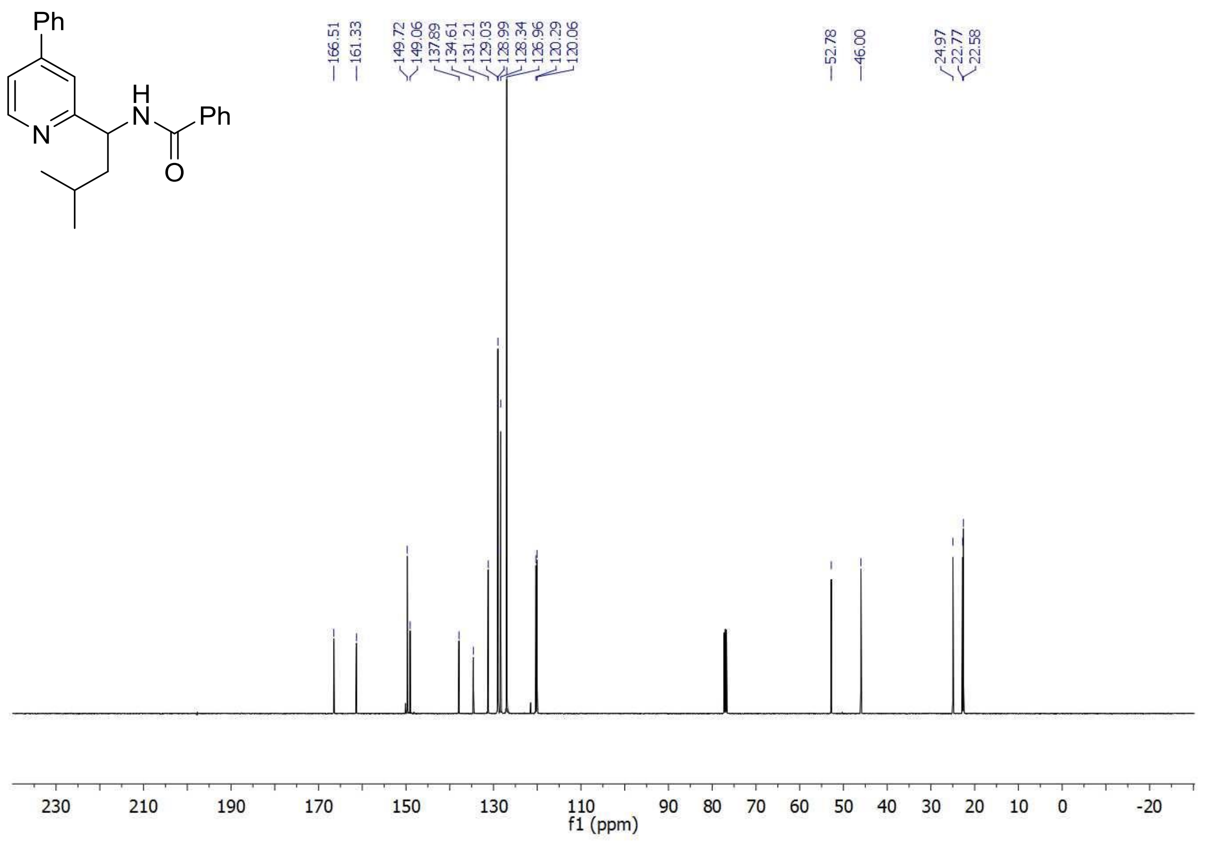

500 MHz $^{1}$H NMR of **8g** in $CDCl_3$

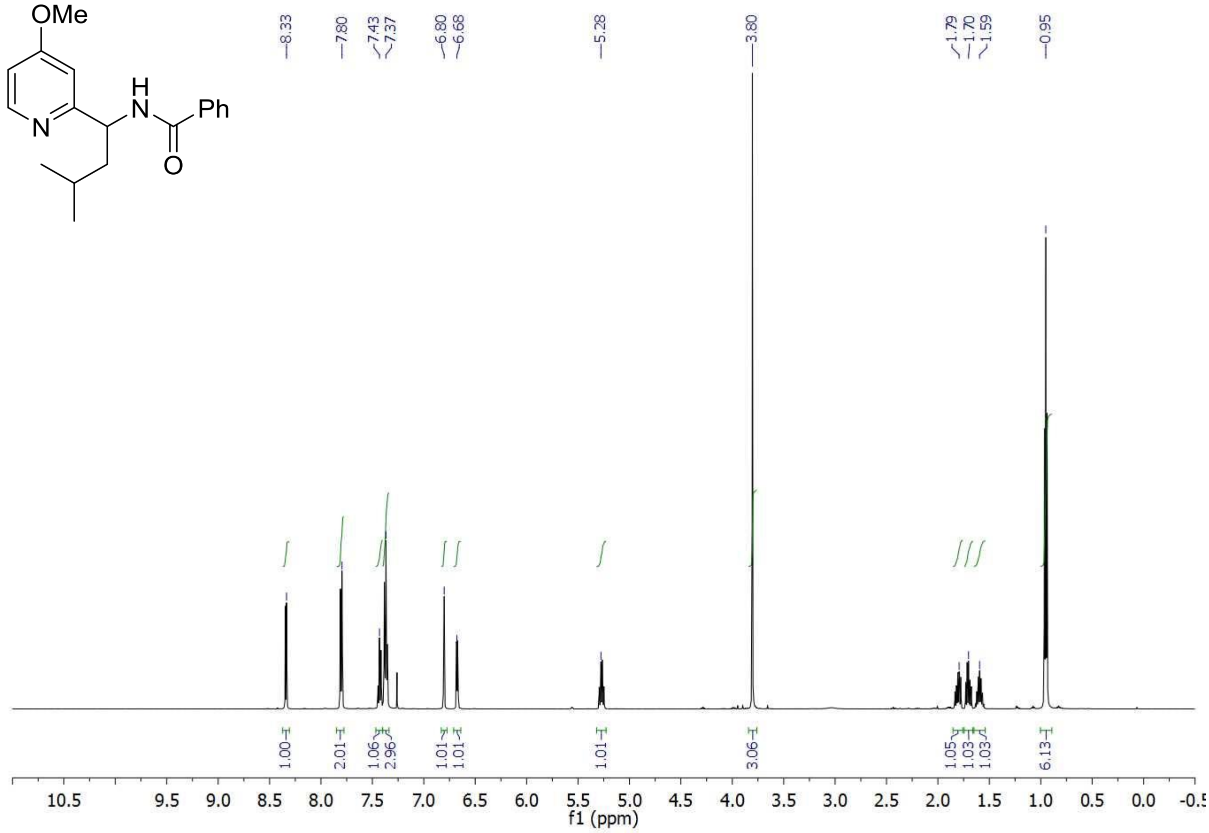


125 MHz $^{13}$C NMR of **8g** in $CDCl_3$

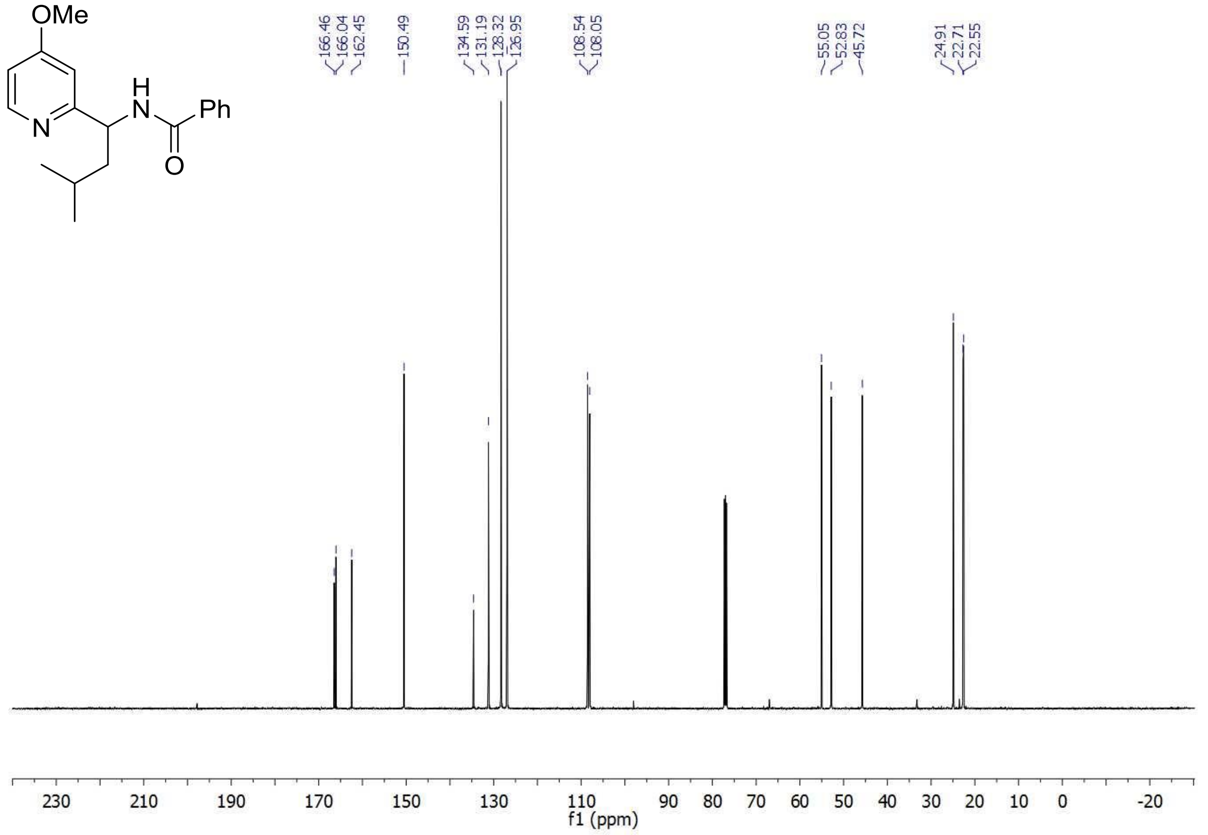

500 MHz $^{1}H$ NMR of **8h** 2-isomer in $CDCl_3$

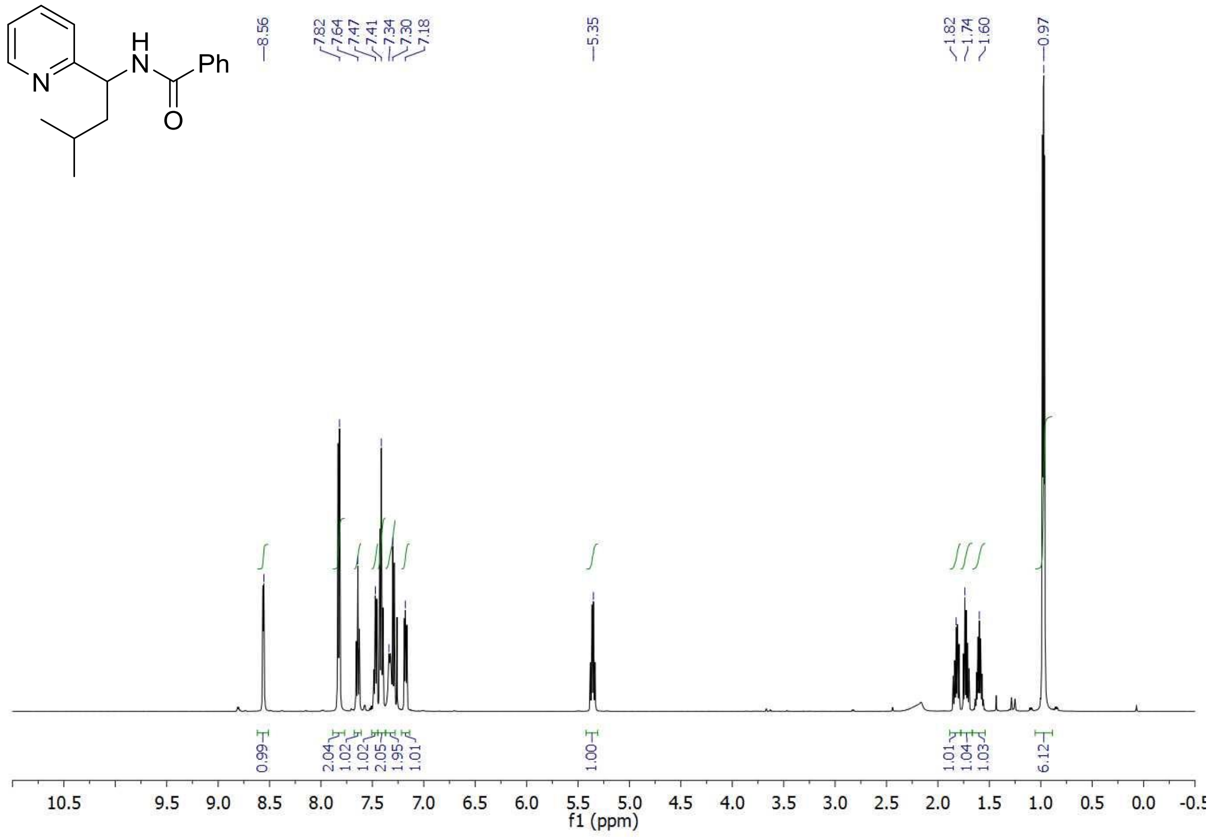


125 MHz $^{13}C$ NMR of **8h** 2-isomer in $CDCl_3$

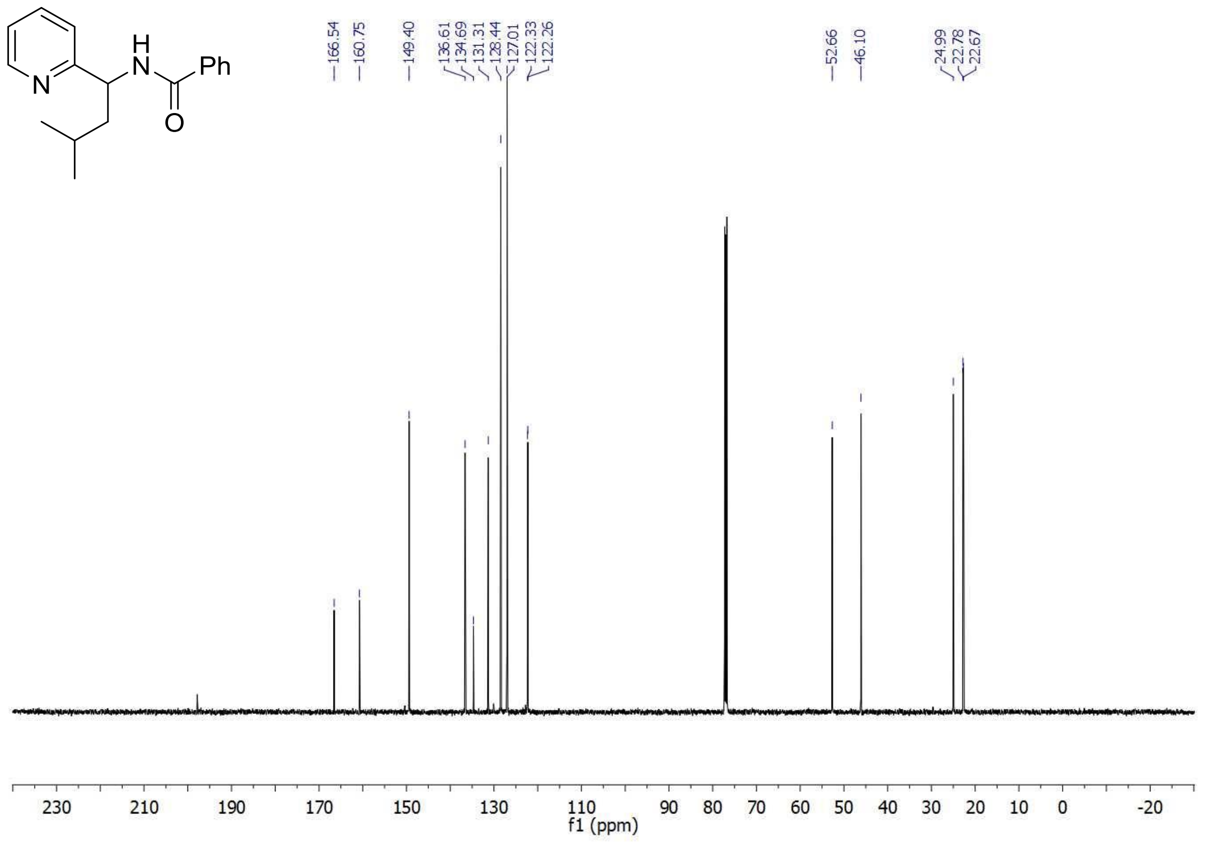

500 MHz $^{1}H$ NMR of **8h** 4-isomer in $CDCl_3$

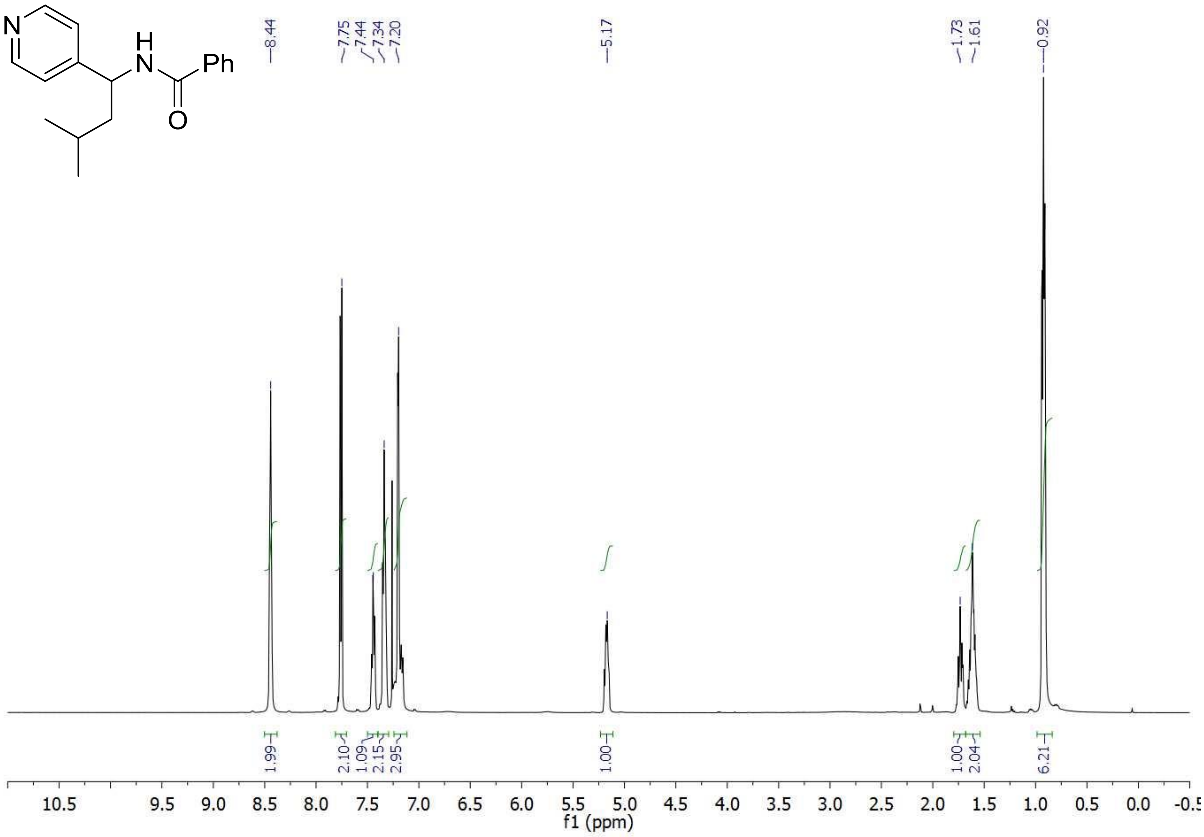


125 MHz $^{13}C$ NMR of **8h** 4-isomer in $CDCl_3$

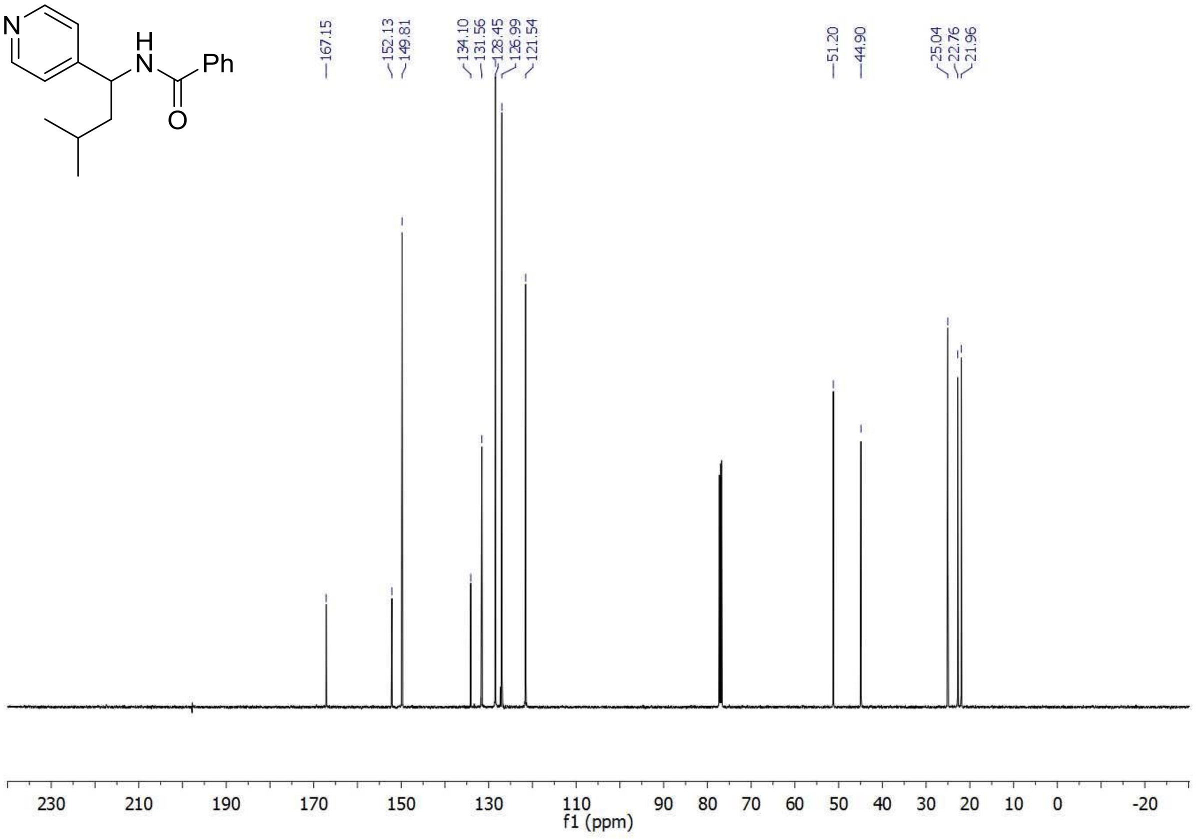

500 MHz $^1$H NMR of **8i** 2,6-isomer in $CDCl_3$

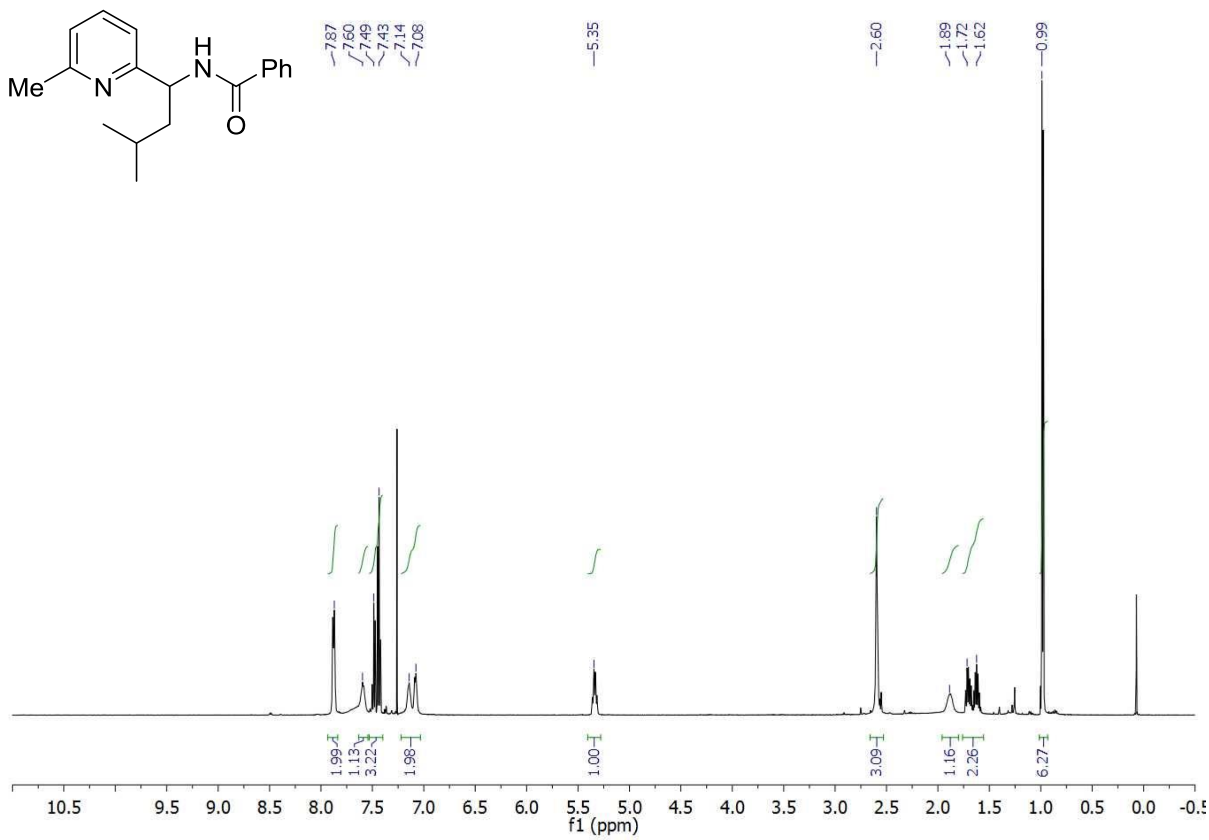


125 MHz $^{13}$C NMR of **8i** 2,6-isomer in $CDCl_3$

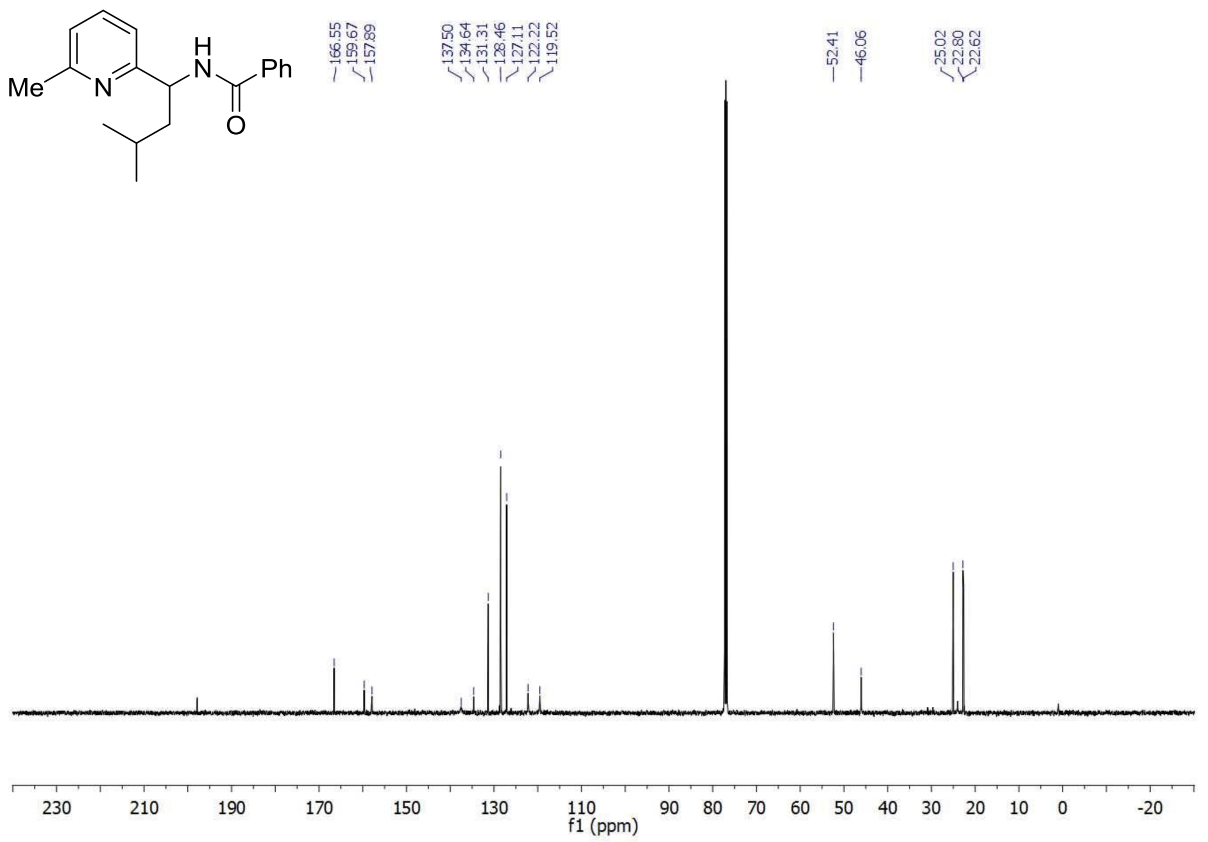

500 MHz $^{1}H$ NMR of **8i** 2,4-isomer in $CDCl_3$

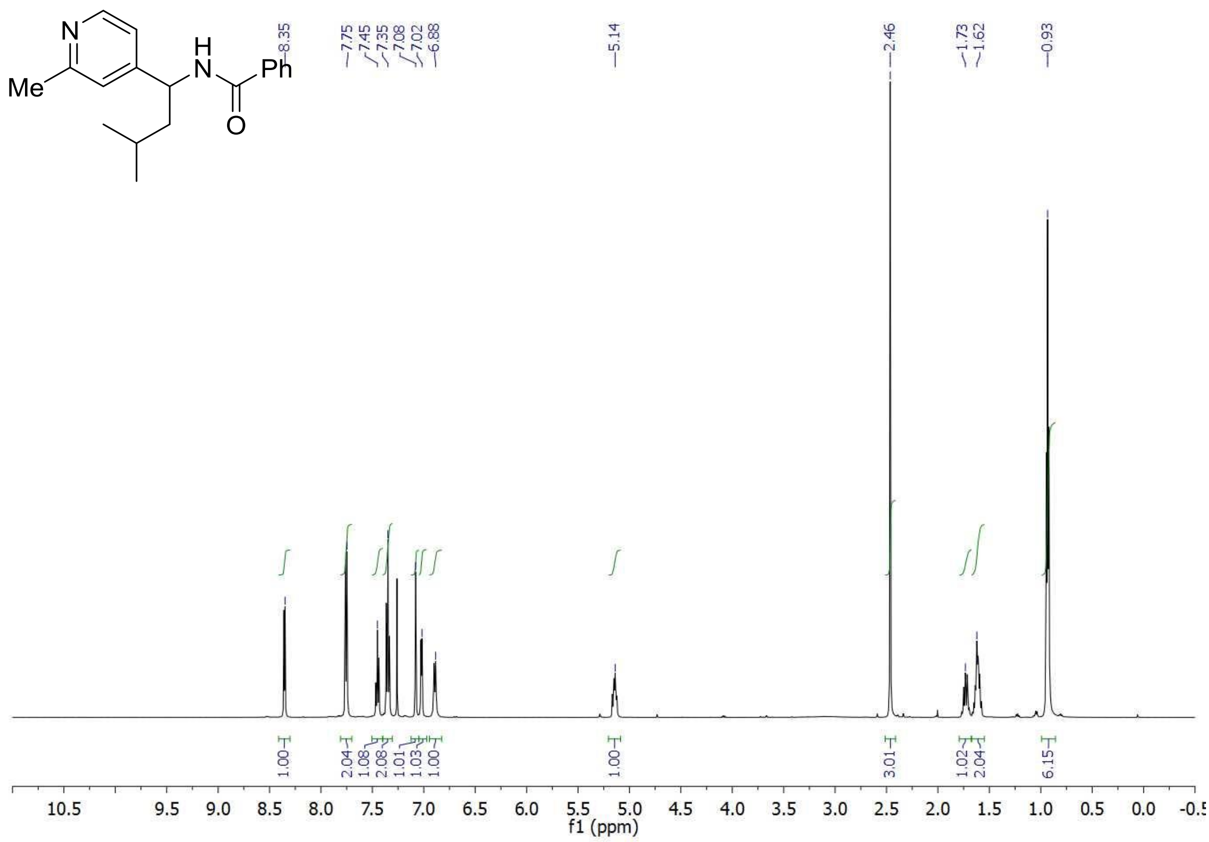


125 MHz $^{13}C$ NMR of **8i** 2,4-isomer in $CDCl_3$

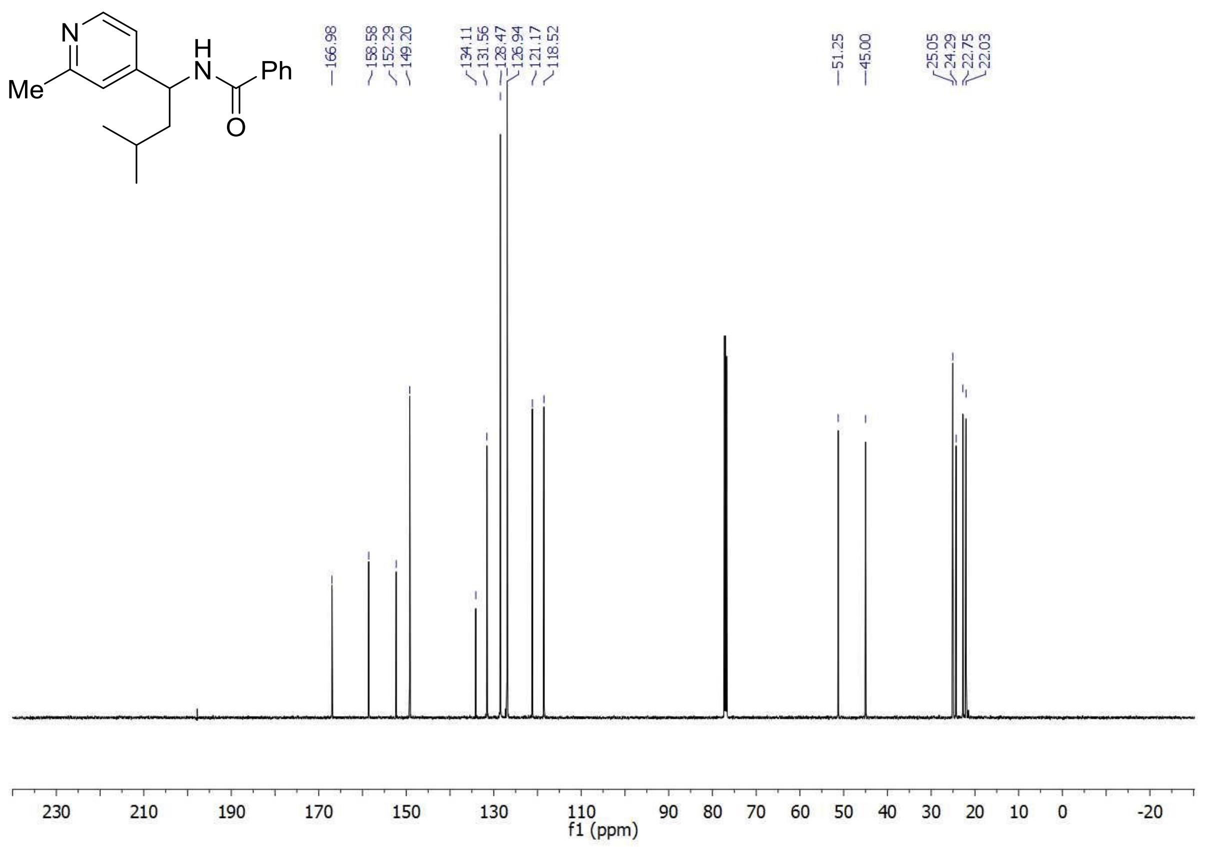

500 MHz $^{1}H$ NMR of **8j** 2,3-isomer in $CDCl_3$

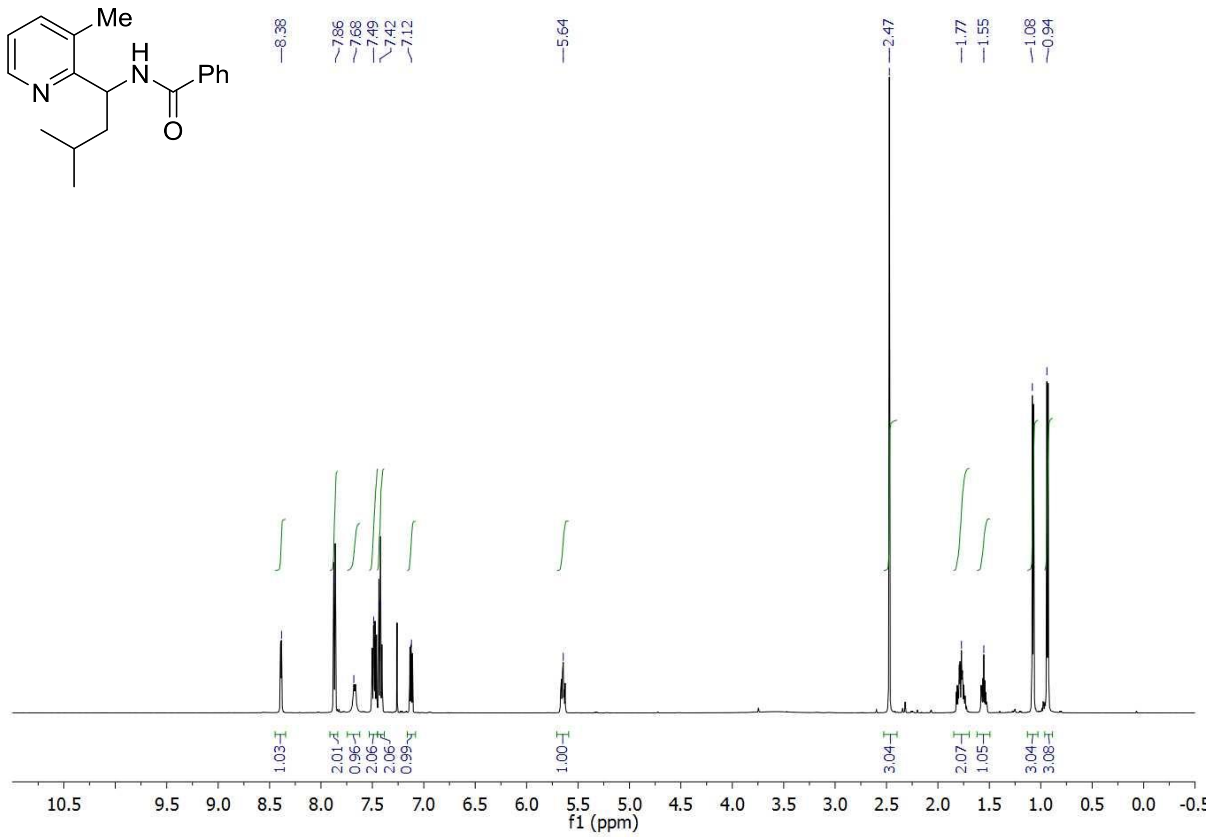


125 MHz $^{13}C$ NMR of **8j** 2,3-isomer in $CDCl_3$

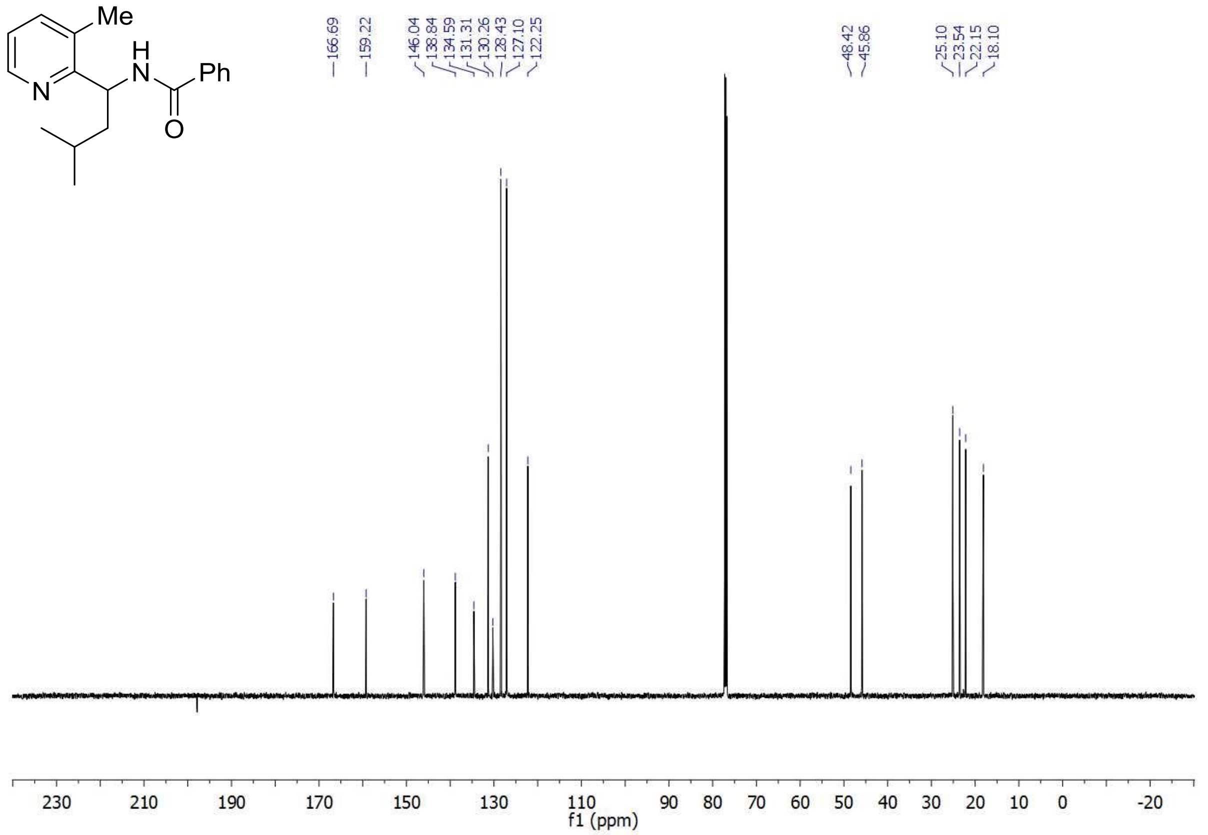

500 MHz $^{1}H$ NMR of **8j** 2,5-isomer in $CDCl_3$

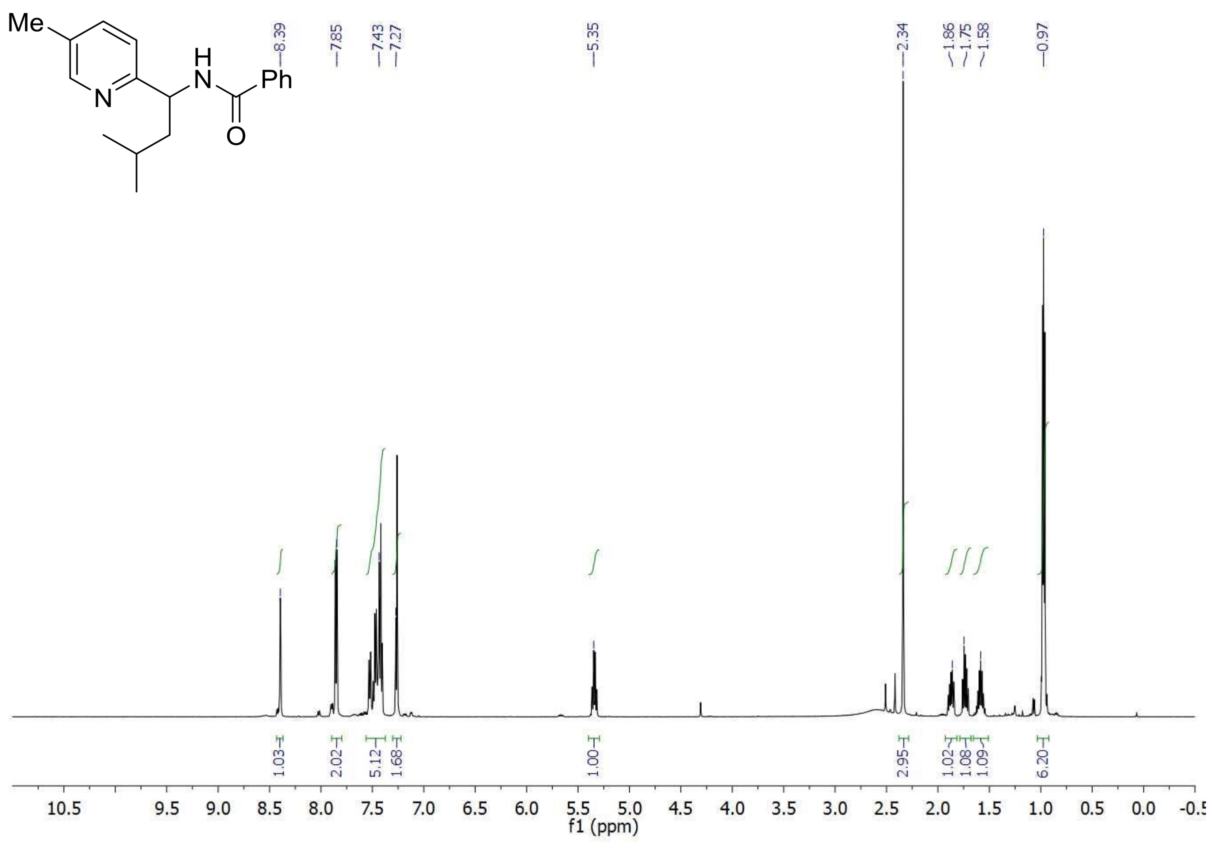


125 MHz $^{13}C$ NMR of **8j** 2,5-isomer in $CDCl_3$

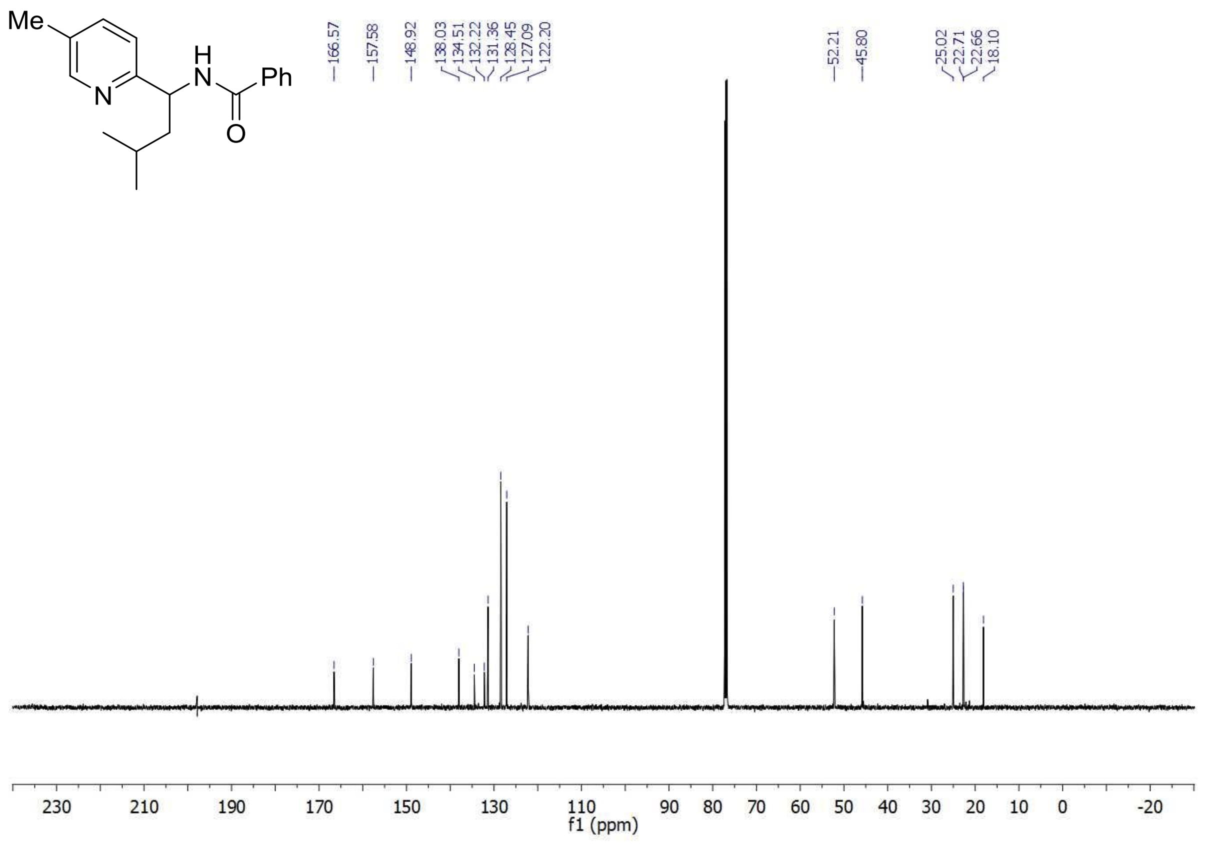

500 MHz $^{1}H$ NMR of **8j** 3,4-isomer in $CDCl_3$

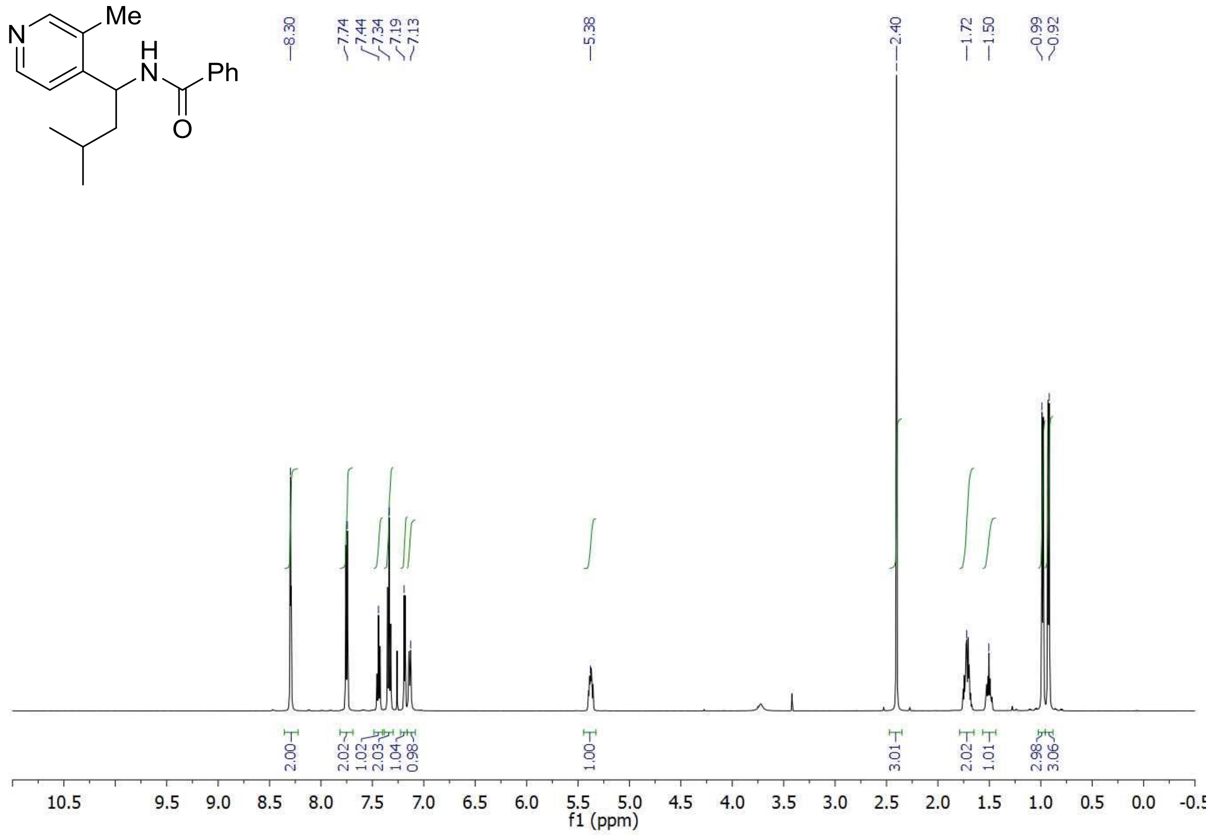


125 MHz $^{13}C$ NMR of **8j** 3,4-isomer in $CDCl_3$

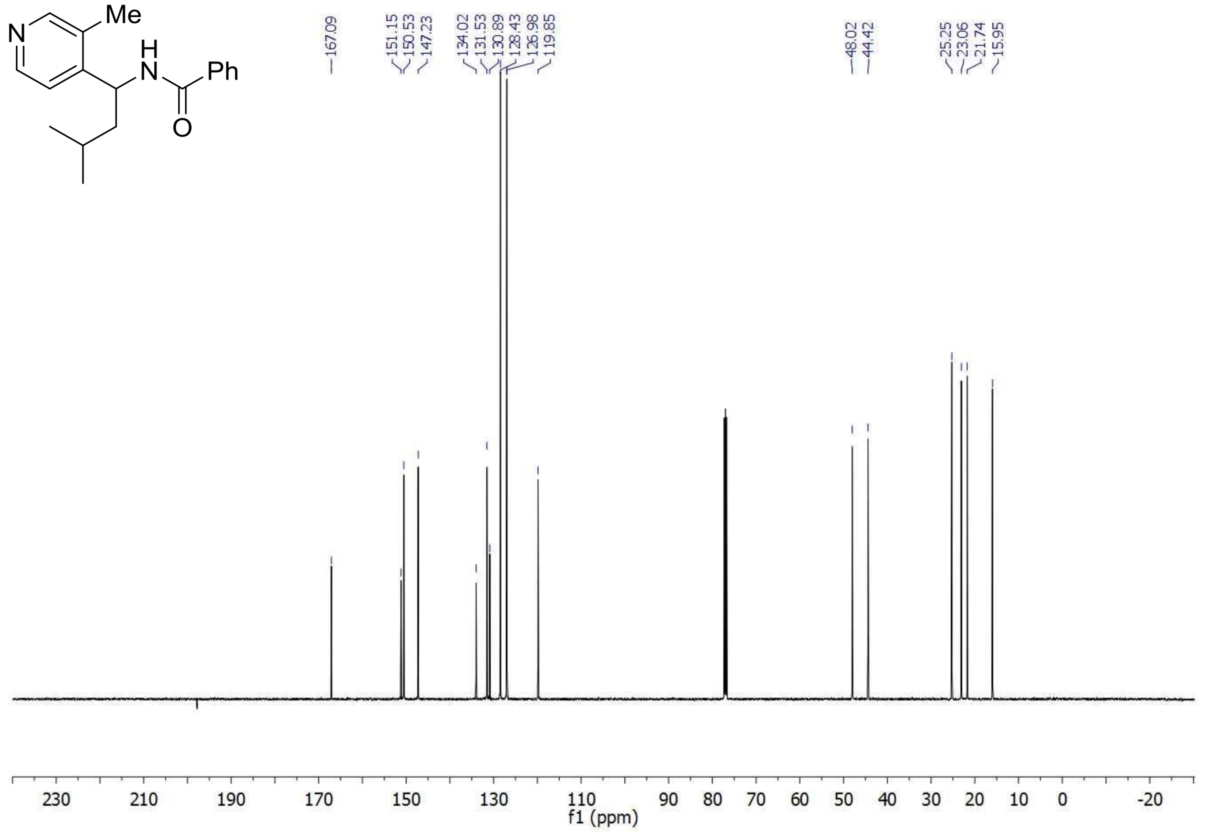

Supplement: File 2 — Copies of spectra for products 8a–j. [file Beilstein_J_Org_Chem-12-01-s002.pdf]
